# Effects of Glass Transition and Structural Relaxation on Crystal Nucleation: Theoretical Description and Model Analysis

**DOI:** 10.3390/e22101098

**Published:** 2020-09-29

**Authors:** Jürn W. P. Schmelzer, Timur V. Tropin, Vladimir M. Fokin, Alexander S. Abyzov, Edgar D. Zanotto

**Affiliations:** 1 Institut für Physik der Universität Rostock, Albert-Einstein-Strasse 23-25, 18059 Rostock, Germany; 2 Frank Laboratory of Neutron Physics, Joint Institute for Nuclear Research, ul. Joliot-Curie 6, 141980 Dubna, Russia; ttv@jinr.ru; 3 Vitreous Materials Laboratory, Department of Materials Engineering, Federal University of São Carlos, UFSCar, São Carlos 13565-905, SP, Brazil; vmfokin@gmail.com (V.M.F.); dedz@ufscar.br (E.D.Z.); 4 National Science Center, Kharkov Institute of Physics and Technology, 61108 Kharkov, Ukraine; alexander.abyzov@gmail.com

**Keywords:** nucleation, crystal growth, general theory of phase transitions, glasses, glass transition, 64.60.Bd General theory of phase transitions, 64.60.Qb ucleation, 81.10.Aj Theory and models of crystal growth, 64.70.kj Glasses, 64.70.Q- T heory and modeling of the glass transition, 65.40.gd Entropy

## Abstract

In the application of classical nucleation theory (CNT) and all other theoretical models of crystallization of liquids and glasses it is always assumed that nucleation proceeds only after the supercooled liquid or the glass have completed structural relaxation processes towards the metastable equilibrium state. Only employing such an assumption, the thermodynamic driving force of crystallization and the surface tension can be determined in the way it is commonly performed. The present paper is devoted to the theoretical treatment of a different situation, when nucleation proceeds concomitantly with structural relaxation. To treat the nucleation kinetics theoretically for such cases, we need adequate expressions for the thermodynamic driving force and the surface tension accounting for the contributions caused by the deviation of the supercooled liquid from metastable equilibrium. In the present paper, such relations are derived. They are expressed via deviations of structural order parameters from their equilibrium values. Relaxation processes result in changes of the structural order parameters with time. As a consequence, the thermodynamic driving force and surface tension, and basic characteristics of crystal nucleation, such as the work of critical cluster formation and the steady-state nucleation rate, also become time-dependent. We show that this scenario may be realized in the vicinity and below the glass transition temperature, and it may occur only if diffusion (controlling nucleation) and viscosity (controlling the alpha-relaxation process) in the liquid decouple. Analytical estimates are illustrated and confirmed by numerical computations for a model system. The theory is successfully applied to the interpretation of experimental data. Several further consequences of this newly developed theoretical treatment are discussed in detail. In line with our previous investigations, we reconfirm that only when the characteristic times of structural relaxation are of similar order of magnitude or longer than the characteristic times of crystal nucleation, elastic stresses evolving in nucleation may significantly affect this process. Advancing the methods of theoretical analysis of elastic stress effects on nucleation, for the first time expressions are derived for the dependence of the surface tension of critical crystallites on elastic stresses. As the result, a comprehensive theoretical description of crystal nucleation accounting appropriately for the effects of deviations of the liquid from the metastable states and of relaxation on crystal nucleation of glass-forming liquids, including the effect of simultaneous stress evolution and stress relaxation on nucleation, is now available. As one of its applications, this theoretical treatment provides a new tool for the explanation of the low-temperature anomaly in nucleation in silicate and polymer glasses (the so-called “breakdown” of CNT at temperatures below the temperature of the maximum steady-state nucleation rate). We show that this anomaly results from much more complex features of crystal nucleation in glasses caused by deviations from metastable equilibrium (resulting in changes of the thermodynamic driving force, the surface tension, and the work of critical cluster formation, in the necessity to account of structural relaxation and stress effects) than assumed so far. If these effects are properly accounted for, then CNT appropriately describes both the initial, the intermediate, and the final states of crystal nucleation.

## 1. Introduction

Despite its long tradition, due to its complexity, the theoretical analysis of crystal nucleation and growth phenomena in glass-forming substances remains an actively developing field of research, with a variety of new topics and applications [1,2,3,4,5,6,7]. In the present paper, we address a particular but very important problem related to the description of the phase transformation kinetics that is specific for crystallization of glass-forming liquids, the effects of the glass transition and of the structural relaxation of the supercooled liquid on the crystallization kinetics in the vicinity and below the glass transition temperature.

The present study of the kinetics of crystallization of glass-forming materials was initiated based on the following considerations. As shown in two preceding papers [8,9], experimental data on the steady-state nucleation rate, $J_{st}$, in crystal nucleation

(1) $$J_{st}=J_{0}\exp\left(-\frac{W_{c}}{k_{B}T}\right),\;W_{c}=\frac{1}{3}\sigma A_{c},\;A_{c}=4\pi R_{c}^{2},\;R_{c}=\frac{2\sigma }{\Delta g},$$

for different glass-forming liquids can be interpreted with high accuracy in terms of classical nucleation theory (CNT) utilizing the Tolman equation [10],

(2) $$\sigma \left(R\right)=\frac{\sigma_{\infty }}{1+\frac{2\delta }{R}},\;\sigma_{\infty }=\sigma_{\infty }\left(T_{m},p_{m}\right),\;\delta =\delta_{\infty }\left(T_{m},p_{m}\right),$$

for the description of the size or curvature dependence of the surface tension, σ(R), of crystallites of critical size. In Equations (1) and (2), $\sigma_{\infty }\left(T_{m},p_{m}\right)$ is the surface tension (referred to the surface of tension) at a planar equilibrium coexistence of liquid and crystal, the so-called Tolman parameter, $\delta_{\infty }\left(T_{m},p_{m}\right)$, determines its change with variations of the radius in a first-order approximation (both quantities, $\sigma_{\infty }$ and $\delta_{\infty }$, are functions of the melting temperature, $T_{m}$, and the corresponding to it melting pressure, $p_{m}$), $W_{c}$ is the work of critical cluster formation, $A_{c}$ and $R_{c}$ are the surface area and the radius (also referred to the surface of tension) of the critical cluster modeled to be of spherical shape (the possibility to treat crystal nucleation employing such model is explained in detail in [8,9]), Δg is the change of the bulk contributions to the Gibbs free energy per unit volume of the crystal phase when the metastable liquid is transformed into the crystalline phase, $k_{B}$ is the Boltzmann constant, and *T* the absolute temperature. Finally, $J_{0}$ reflects the kinetic mechanism of cluster formation and growth.

**Figure 1:**
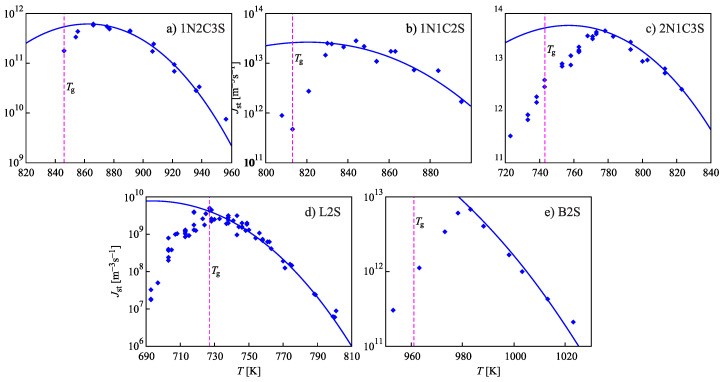
Steady-state nucleation rates of several glass-forming melts [(**a**) ${\mathrm{Na}}_{2}O\cdot 2\mathrm{CaO}\cdot 3{\mathrm{SiO}}_{2}$ (1N2C3S), (**b**) $22.4{\mathrm{Na}}_{2}O\cdot 28.0\mathrm{CaO}\cdot 49.6{\mathrm{SiO}}_{2}$ (1N1C2S), (**c**) $2{\mathrm{Na}}_{2}O\cdot 1\mathrm{CaO}\cdot 3{\mathrm{SiO}}_{2}$ (2N1C3S), (**d**) ${\mathrm{Li}}_{2}O\cdot 2{\mathrm{SiO}}_{2}$ (L2S), (**e**) $\mathrm{BaO}\cdot 2{\mathrm{SiO}}_{2}$ (B2S)] utilizing the Tolman equation. The parameters and the sources of the nucleation rate data are given in [8,9]. By $T_{g}$, the glass transition temperature is specified according to the definition proposed first by Tammann (see text).

In [8,9], $\sigma_{\infty }$ and δ were taken as fit parameters to reach an agreement with the experimental nucleation rates. In addition, theoretical estimates for the Tolman parameter have been developed based on a generalization of the Stefan–Skapski–Turnbull rule [11,12]. These theoretical estimates are shown to be in good agreement with the values obtained by mentioned fitting procedure. Some of these results are presented in Figure 1. It is evident that, when assigning appropriate values to the parameters $\sigma_{\infty }$ and δ, the Tolman equation allows a good description of the experimental data down to the temperatures, $T_{max}$, corresponding to the maximum of the steady-state nucleation rate, $J_{st}\left(T_{max}\right)$. However, a serious problem remains unresolved which is denoted frequently as the “breakdown of CNT” for $T⪅T_{max}$. To reach an agreement between theory and experiment for lower temperatures $T⪅T_{max}$, additional factors affecting crystal nucleation have to be accounted for going beyond the standard approach commonly employed in CNT.

The origin of deviations of theoretical steady-state nucleation rates from experimental data for temperatures below $T_{max}$ is a matter of continuous debate. This breakdown problem also cannot be resolved by the account of self-consistency corrections to the work of critical cluster formation as demonstrated in our recent analysis [13]. As shown there, when self-consistency corrections are introduced into the theory, the Tolman equation supplies us again with an appropriate description of the dependence of the nucleation rate on temperature, but, similarly to the results shown in Figure 1, only down to $T_{max}$ and not in the range $T⪅T_{max}$. Consequently, in the temperature range below $T_{max}$, additional features have to be incorporated into the model of crystal nucleation also in such more correct self-consistent treatment to arrive at an agreement of theory and experiment.

**Figure 2:**
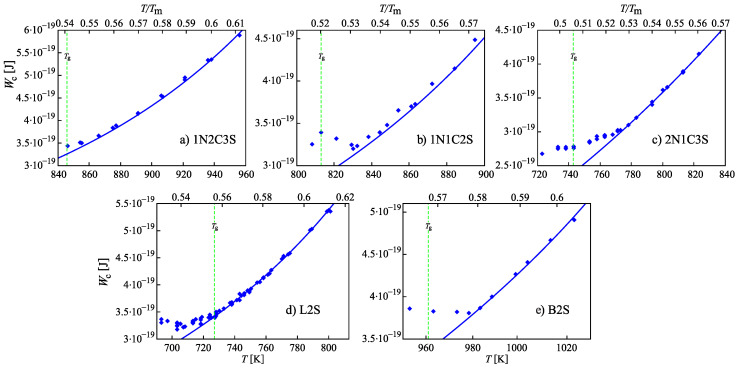
Thermodynamic barrier for crystal nucleation, $W_{c}/k_{B}T$, versus temperature for a variety of glass-forming liquids (the same liquids as shown in Figure 1) in dependence on temperature, *T*, and reduced temperature, $T/T_{m}$. $T_{m}$ is the melting temperature. (**a**) 1N2C3S, (**b**) 1N1C2S. (**c**) 2N1C3S, (**d**), L2S, (**e**) B2S.

In interpreting the steady-state nucleation rates shown in Figure 1 in standard terms of CNT, a behavior of the work of critical cluster formation on temperature would have to be assumed as shown in Figure 2. At temperatures above $T≅T_{max}$, the work of critical cluster formation, $W_{c}$, decreases with decreasing temperatures as expected based on CNT. However, for temperatures below $T⪅T_{max}$, $W_{c}$ has to increase again for a proper interpretation of the experimental data. Such behavior cannot be understood in terms of standard concepts of CNT. The type of behavior as shown in Figure 2 was observed long ago in [14,15]. At that time, only a few examples of glass-forming liquids exhibiting such behavior were known; hence, they were considered to be anomalous exceptions. Meanwhile such kind of behavior was found to be a general feature of crystal nucleation in glass-forming liquids. Consequently, its correct understanding is of major importance in the formulation of a comprehensive theory of these processes.

The following additional factors that could eventually provide an explanation of these deviations of theoretical predictions from experimental data have been analyzed by us in preceding papers: (i) non-monotonic dependence of the surface tension on the size of the critical crystal nucleus [14,15,16,17,18,19], (ii) interplay of evolution of elastic stresses and stress relaxation in crystal nucleation [19,20,21,22,23,24,25,26], (iii) variation of the size of the structural units responsible for crystallization [27], (iv) effect of spatially heterogeneous structure of glass-forming liquids on crystal nucleation [28,29], (v) deviations of the bulk state parameters of the critical clusters from the respective macroscopic properties of the newly evolving crystalline phases [21,30,31,32,33,34,35,36]. Further detailed analysis of these suggestions and their implications is of high interest.

However, in the present paper we would like to follow another approach. It was suggested as a possible way of resolution of described above problem briefly already in [13,26,33,34,35,37]. Its further advancement was stimulated by comprehensive experimental investigations of the crystal nucleation characteristics of glass-forming liquids at low temperatures performing them over much more prolonged periods of time as it is commonly done in similar studies. They will be presented in detail in [38]. In this treatment, mentioned peculiarities in crystallization at temperatures below $T_{max}$ are correlated with the glass transition. More specifically, we study the effects on crystal nucleation resulting from deviations of the supercooled liquids from its metastable equilibrium state and of the relaxation of the liquid to this state. We will rely here on the general concepts developed first in [37] and further advance this approach.

First, we will show how deviations of the supercooled liquid from the corresponding metastable state affect the thermodynamic driving force of crystallization and the surface tension. This analysis is then supplemented by the specification of the conditions at which relaxation of the glass-forming liquid proceeds more slowly as compared to nucleation-growth processes. In line with earlier investigations it is reconfirmed, in addition, that only at such conditions, when the characteristic times of relaxation are longer than the characteristic times of crystal nucleation, elastic stresses evolving in nucleation may significantly affect this process. Extending mentioned previous analysis, relations are derived for the dependence of the surface tension of critical crystallites on elastic stresses. An illustration of the analytical results will be presented then performing a quantitative treatment of crystal nucleation in a slowly relaxing liquid for a model system developed and widely employed in the description of the properties of glass-forming liquids and the glass transition [20,21]. In the model approach, we will not include the possible dependence of the kinetic parameters like viscosity, diffusion coefficient, and structural relaxation time on the degree of deviation of the liquid from metastable equilibrium. This topic will be addressed in a future study, it will qualitatively not change the main conclusions derived in the present analysis.

Two previously performed studies on the CNT-breakdown, partly including results of experiments performed over prolonged times, can be traced in [39,40]. In both studies it was concluded that the above discussed deviations of theoretical steady-state nucleation rates from experimental data for temperatures below the temperature of the maximum steady-state nucleation rates are largely caused by the use of nucleation rate data for processes that have not yet reached the ultimate steady-state at the respective nucleation temperatures. Based on the theory developed, here a much more comprehensive interpretation is advanced: (i) experimental results on the nucleation rates as shown in Figure 1 are the temporary steady-state nucleation rates established for the respective glass-forming melts after a time-lag in nucleation, as described in CNT. Both steady-state nucleation rate and time-lag depend on the initial state of the liquid and, in particular, on the degree of its deviations from metastable equilibrium. Experiments and CNT are in full agreement if such deviations from equilibrium are properly accounted for. (ii) Structural relaxation processes of the glass-forming liquid to the metastable equilibrium state result in slow variations of the temporary steady-state nucleation rates. (iii) The ultimate steady-state nucleation regime is established only when the structural order parameters have approached their equilibrium values. We show that the long times necessary to reach the ultimate steady-state regime at temperatures below $T_{g}$ are not just the classical nucleation time lags (discussed in CNT assuming that the liquid has reached a metastable state prior to intensive nucleation), instead they are predominantly related to the structural relaxation of the glass. For very low temperatures, this ultimate steady-state may not be reached at all in realistic measurement times. (iv) As a rule, elastic stresses evolving in crystal nucleation will affect the steady-state nucleation rates for temperatures below the nucleation rate maximum. They will lead to deviations from the curves describing experimental data employing the Tolman equation. In this way, the present paper provides an alternative, more detailed interpretation of the problem of “breakdown of CNT”: If deviations of the structural order parameters of the glass-forming liquids from their metastable equilibrium values and their relaxation processes are properly accounted for, then CNT appropriately describes both the initial, the intermediate, and the final states of crystal nucleation.

The paper is structured as follows. The basic ideas of the theoretical description of crystal nucleation accounting for the effects of deviations of the state of the liquid from metastable equilibrium and relaxation processes to this particular state are outlined in Section 2. In Section 3 it is shown how deviations of the liquid from its metastable equilibrium affect the thermodynamic driving force of crystallization and the surface tension. Whether or not such deviations may result in changes of the crystal nucleation kinetics depends on the ratio of the characteristic time scales of relaxation and crystal nucleation. As it turns out these effects become of significant importance at temperatures in the vicinity of the glass transition temperature and below it. This topic is addressed in Section 4. The influence of the interplay of elastic stress evolution and stress relaxation on nucleation is analyzed in Section 5. A discussion of the results including a brief comparison with experimental data confirming them (Section 6) and a summary of the conclusions (Section 7) complete the paper.

## 2. The Model: Basic Assumptions

In CNT, it is commonly assumed that crystal nucleation starts only after the liquid or the glass have reached its metastable equilibrium state [20,21,41]. Only with such assumption, the thermodynamic driving force of crystallization and the surface tension can be determined in the way as it is commonly performed in CNT. In particular, the thermodynamic driving force of crystallization is connected with the change of the Gibbs free energy per unit volume of the crystal phase in the transformation of the metastable liquid to the crystal. The surface tension is assumed to be equal to the respective value of a critical crystallite in the metastable liquid and determined by indirect measurements, computer simulations or taken as a fit parameter to arrive at an agreement between theory and experiment. Utilizing CNT and employing the capillarity approximation and the Stokes–Einstein–Eyring equation (that relates the diffusion coefficient to the viscosity of the liquid), such approach can be confirmed theoretically [20,21,42] and also by a variety of experimental data [43,44,45,46,47]. However, both assumptions are known to be in general not correct. The Stokes–Einstein–Eyring equation is employed in glass science since it is much easier to determine the viscosity as compared to the effective diffusion coefficients governing nucleation and growth. Its degree of validity is discussed for decades and decoupling of diffusion and viscosity near to $T_{g}$ is well-known to be a general feature. The capillarity approximation is the simplest approach for the specification of surface contributions to the Gibbs free energy in crystal cluster formation. However, its application leads to serious problems in the theoretical description.

Going beyond the capillarity approximation and the Stokes–Einstein–Eyring equation, the situation becomes different [48,49,50]. In [26,33,35], a review of these non-trivial problems is given, and general theoretical considerations are outlined showing that near and below the glass transition range the opposite situation can be realized, structural relaxation processes may proceed slowly as compared with crystal nucleation. In such cases, the nucleation kinetics can be significantly affected by structural relaxation processes proceeding in the glass-forming melt as discussed from an experimental point of view in [2,51,52,53,54,55,56,57,58,59,60]. They result in changes of the state of the liquid in nucleation. In addition, the long times required for complete relaxation can be the origin of the possible existence of precursor structures in the liquid significantly affecting crystal nucleation (denoted in polymer physics as self-nucleation). The relevance of such situation in polymer crystallization is caused by the following circumstances [26,61,62]: extended chain configurations of polymer chains, representing the equilibrium configuration in the crystalline state, are commonly not realized for long-chain polymers because of entropic penalties. Polymer crystals generally represent non-equilibrium states usually referred to as folded chain crystals. Only crystals containing fully stretched chains can be regarded as being in an equilibrium thermodynamic state. The occurrence of non-equilibrium folded states has its origin in the high internal conformational entropy of individual chains in the melt. Sommer and Reiter [61] made an estimate for the time needed to create a fully stretched chain made of 100 monomers by spontaneous fluctuations. The required time of $10^{58}$ s is obviously beyond any practical relevance.

However, as we will show here, deviations from metastable equilibrium occur also as a rule in crystallization of silicate glasses at low temperatures. Direct experimental data in support of the point of view that nucleation may proceed in such systems concomitantly with relaxation are presented in an accompanying paper [38]. Some of these experimental data will be shown here in Section 6. In the present paper, we will reconfirm this point of view by proposing and analyzing a simple theoretical model describing the effects of glass transition and relaxation of the supercooled liquid on crystal nucleation. Note also that similar effects have been recently found to be of significant importance in the study of atmospheric aerosols, in particular, in analyzing the effect of chemical aging of aqueous organic aerosols on the rate of their steady-state nucleation [63].

One strong argument in support of the explanation of the “breakdown” of CNT advanced here consists in the following considerations. Adopting the basic ideas in the interpretation of the physical nature of glasses and the glass transition as developed long ago by Simon, Tammann, de Donder, Leontovich and Mandelstam, Kauzmann, Davies and Jones, Tool, Prigogine and Defay, Bartenev, Volkenstein and Ptizyn and many others [20,21,48,49,50,64,65,66], near to the glass transition temperature (in the glass transition region), the metastable liquid is transformed into a frozen-in non-equilibrium state, the glass. In the thermodynamic description, structural order parameters, $\left\{\xi_{i}\right\}$, have to be introduced to account for such effects. They are required to describe the deviations from metastable equilibrium. In the course of the glass transition, they are freezing-in, i.e., becoming independent of time. Their behavior reflects the well-known feature of the glass transition that the state of the liquid, respectively, the glass becomes dependent on the cooling rate or the prehistory of the liquid. These deviations from metastable equilibrium result in additional contributions to the thermodynamic driving force and the surface tension as compared to the common treatment in CNT. They have to be incorporated, consequently, into the theoretical description to be able to appropriately describe experimental data.

For that purpose, in the present analysis we utilize and generalize relations obtained from a simple lattice-hole model of liquids derived and employed for the interpretation of the properties of glasses. The model is described in detail in [20,21,49,67,68,69]. A similar model has been used by Johari [70] in the analysis of the problem of configurational and residual entropies of non-ergodic crystals and their entropy’s behavior on glass formation. Details of the model can be traced in cited references, some of its consequences are briefly reviewed in the Appendix A as far as they are required for the present analysis. Here we will employ directly some of the main results of these studies.

In particular, the dependence of the metastable equilibrium value, $\xi_{e}$, of the structural order parameter, ξ, on temperature is given by

(3) $$\frac{{\left(1-\xi_{e}\left(T\right)\right)}^{2}}{\ln\xi_{e}\left(T\right)}=-\frac{1}{\chi }\left(\frac{T}{T_{m}}\right).$$

At the melting or liquidus temperature, $T=T_{m}$, the value of $\xi_{e}$ should be approximately equal to $\xi_{e}\left(T_{m}\right)=0.05$ (corresponding to experimentally observed density differences between liquid and crystal at the melting temperature [20,21]). In the computations, here we set χ=3.32 for the realization of this condition [67,68,69].

A wealth of experimental data shows that the relaxation kinetics of glass-forming melts to the respective metastable equilibrium states can be appropriately expressed in the form [20,21,37,71]

(4) $$\frac{d\xi }{dt}=-\frac{1}{\tau_{R}\left(T,p,\xi \right)}\left(\xi -\xi_{e}\right),$$

where the relaxation time $\tau_{R}\left(T,p,\xi \right)$ is a function of pressure, temperature and, in general, of the set of structural order parameters. Here we assume that one structural order parameter is sufficient for the description. Even with such an assumption, as shown in [37,71], Equation (4) allows us to understand the origin of stretched exponential type relaxation processes and results in estimates of the coefficients in the respective relaxation law in agreement with experimental findings.

In the numerical computations, we start the cooling process at $T=T_{m}$. We cool the system with a with a constant rate,

(5) $$q=\frac{dT}{dt}=\mathrm{const},$$

down to the temperature where nucleation processes are studied. Since, dT and dt are linearly dependent, we can always go over from a differentiation or integration with respect to time to the respective procedures with respect to temperature (dT=qdt) and vice versa (dt=dT/q) with different signs of the parameter *q* (q<0 for cooling and q>0 for heating processes). Equation (4) then takes the form

(6) $$\frac{d\xi }{dT}=-\frac{1}{q\tau_{R}}\left(\xi -\xi_{e}\right).$$

Solving this equation, we can determine ξ(T) for any desired temperature, $T\leq T_{m}$, in the range of metastable initial states of the liquid.

In the computations, we employ expressions for the relaxation time of the form

(7) $$\tau_{R}=\tau_{0}\exp\left(\frac{U_{a}}{RT}\right),\;U_{a}=U_{a}^{*}\left(\frac{T}{T-T_{0}}\right),\;\tau_{0}=\frac{h}{k_{B}T}$$

with

(8) $$T_{0}=\frac{T_{m}}{2},\;\frac{U_{a}^{*}}{RT_{g}^{\left(T\right)}}=7.5,\;\frac{T_{g}^{\left(T\right)}}{T_{m}}=\frac{2}{3},$$

as described in detail in [67,68,69]. Here $U_{a}$ is the activation energy for the structural relaxation processes considered, *h* is Planck’s constant, $T_{g}^{\left(T\right)}$ is the glass transition temperature defined in accordance with the suggestion by Tammann [72] correlating it with a Newtonian viscosity, η, equal to $\eta \left(T\right)≅10^{12}$ Pa s at $T=T_{g}^{\left(T\right)}$. These relations result in

(9) $$\tau_{R}=\tau_{0}\exp\left(7.5\frac{T_{g}^{\left(T\right)}}{T-T_{0}}\right),\;\tau_{0}=\frac{h}{k_{B}T}.$$

The choice of the parameter $U_{a}^{*}$ is motivated by the following considerations. As noticed first by Kauzmann [73], for typical cooling rates the glass transition temperature of a variety of silicate glass-forming melts is described by a Gaussian distribution with the average near to $T_{g}^{\left(T\right)}≅\left(2/3\right)T_{m}$. In the specification of the expressions for the relaxation time, we assume that this relation holds for the model system under consideration.

In general, the maximum of the steady-state nucleation rate is determined by the interplay of increase of viscosity and decrease of the work of critical cluster formation. The values of the steady-state nucleation rate at $T_{max}$ decrease considerably with increasing ratio $T_{max}/T_{m}$ [74]. For this reason, experimental data on bulk crystal nucleation in the systems under consideration are easier to observe experimentally at temperatures corresponding to the lower part of mentioned above distribution of $T_{g}$-values, i.e., for glass-forming melts with a glass transition temperature lower than the average value $T_{g}^{\left(T\right)}≅\left(2/3\right)T_{m}$. This particular feature of the crystal nucleation kinetics is also reflected in Figure 1 and Figure 2.

**Figure 3:**
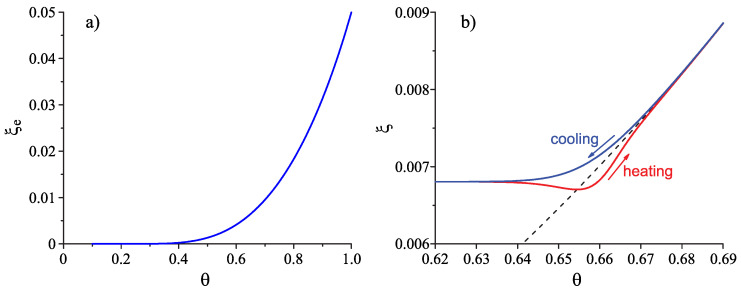
Structural order parameter, ξ, and its equilibrium value, $\xi_{e}$, in dependence on reduced temperature, $\theta =T/T_{m}$. (**a**) Dependence of the equilibrium value of the structural order parameter for the whole range of temperatures between melting or liquidus temperature, $T_{m}$, and absolute zero as obtained in the framework of the lattice model employed here. An outline of the basic ideas of this model and consequences can be found in [20,21,49,67,68,69]. (**b**) Typical behavior of the structural order parameter, ξ, in dependence on temperature in the vicinity of the glass transition range if the liquid is cooled down and heated with the same constant rate of change of temperature. The dependencies ξ(T) are shown by full curves if the system is cooled down (blue curve) and heated (red curve) with a constant rate (here taken equal to (dT/dt)=1.3 K/s or $\left(d\theta /dt\right)=10^{-3}s^{-1}$), the dashed curve shows the equilibrium value of this parameter in the given range of temperature.

In the simplest approach used here we neglect the dependence of $\tau_{R}$ on the structural order parameter. Generalizations will be considered in further extensions of the present model utilizing methods advanced in [20,21,22,23,37,71,75,76,77,78] and briefly discussed below. The behavior of the structural order parameter on cooling and heating with a moderate constant rate of change of temperature is illustrated for this model in Figure 3.

The glass transition temperature, $T_{g}$, depends, in general, on cooling and heating rates [20,21,64,66]. However, we consider here processes of cooling of the liquid at typical laboratory time-scales employed in the preparation of the samples for a detailed study of nucleation phenomena. At such moderate cooling rates, the glass transition temperature can be defined in line with the proposal by Tammann [72]. The important point, the noted above very strong argument in support of the approach to the resolution of the problem of the “breakdown” of CNT advanced here, is that, at these process conditions, the glass transition temperature and the temperature of the maximum of the crystal nucleation rate, $T_{max}$ and $T_{g}^{\left(T\right)}$, are directly correlated, they are close to each other, $T_{max}≊T_{g}^{\left(T\right)}$ [30,43,44,45,79,80]. Consequently, the specific features occurring at the glass transition temperature (Figure 3) may be the origin for the problems in the theoretical interpretation of experiments on measurements of steady-state nucleation rate illustrated in Figure 1.

In the present paper, we present a confirmation of these ideas based on the analysis of a particular model. To proceed in this direction, we have to solve in the next step the problem of how these specific features have to be incorporated into the description of crystal nucleation.

## 3. Implications for the Description of Crystal Nucleation: Thermodynamic Aspects

**Figure 4:**
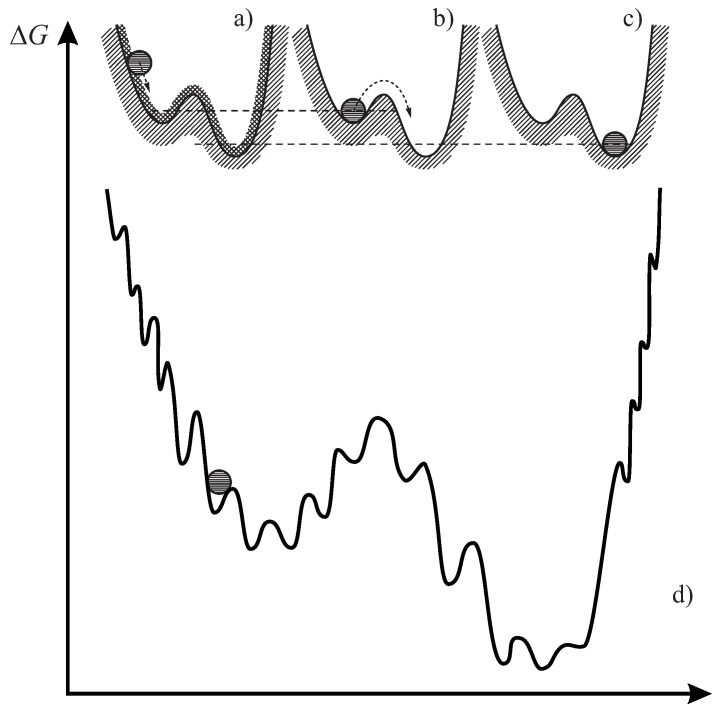
Mechanical analogy for the interpretation of the differences between (**a**) the glass, (**b**) the metastable liquid, and (**c**) the stable crystalline state ($T<T_{m}$) as proposed by Simon. In (**d**), a modification of Simon’s picture of the vitreous state and its relation to the crystalline state is given accounting for the potential energy landscape model of glass-forming systems as advanced by Goldstein [81] (for more details, see Section 2 in [20,21], text and [50] where the figure is taken from).

As already noted in the introduction, in CNT it is assumed that the liquid has reached its metastable equilibrium state first and only after this process is completed, nucleation occurs. In such cases, we can employ in the description of crystal nucleation values of the thermodynamic driving force and the surface tension obtained considering critical crystallites to be formed in a metastable liquid. In terms of the model considerations discussed in Section 2, this condition implies that the structural order parameter, ξ, has reached its equilibrium value, $\xi =\xi_{e}$. This path of evolution corresponds to the situation illustrated in Figure 4b by a mechanical analogy of the motion of a particle in a force field, as proposed by Simon. The equilibrium state of the liquid corresponds to a local minimum of the Gibbs’ free energy. It is a thermodynamically metastable state since it can be transformed into a crystal corresponding to lower values of the Gibbs’ free energy per unit mass of the crystal phase. In this process, a barrier has to be overcome corresponding to the work of critical cluster formation.

However, on cooling the liquid from the melting or liquidus temperature, $T_{m}$, down to temperatures $T≲T_{g}^{\left(T\right)}$, the thermodynamic driving force and the surface tension may become dependent not only on temperature, pressure, and composition of the liquid, but also on the value of the structural order parameter, ξ, since the liquid is transferred into a non-equilibrium state with a value of the structural order parameter, ξ, not corresponding to metastable equilibrium, $\xi_{e}$. As illustrated in Figure 4a, such dependence has to be accounted for near and below the glass transition temperature, $T_{g}^{\left(T\right)}$, i.e., at $T≲T_{g}^{\left(T\right)}$. Simon’s model was slightly modified by us accounting for potential energy landscape approaches advanced by Goldstein (see [20,21,50,81]). Already in the approach to the metastable equilibrium state, the liquid can be trapped temporarily in some local minima determined by the structure of the liquid (Figure 4d). To continue relaxation, another potential barrier has to be overcome by thermal fluctuations. As is well-known, in particular, from polymer physics, different crystalline phases may be formed in the course of nucleation and growth processes Figure 4d (e.g., [61,62]). Consequently, such local minima may occur also in the evolution to the crystal phase corresponding to the right part of the model curve in Figure 4d.

Such peculiar effects cannot be described by the relaxation model based on the thermodynamics of irreversible processes used here. It supplies us with the theoretical description of the general trends of evolution but, due to its simplicity, it does not cover such additional but, of course, also possible peculiar details. However, in both cases, independent on whether the liquid evolves towards metastable equilibrium or is trapped temporarily in a potential energy minimum, as described in Figure 4a,d, we have to specify how deviations of the state of the liquid from metastable equilibrium affect both the thermodynamic driving force and the surface tension. In terms of the thermodynamic description, we have to determine how these basic quantities of nucleation theory depend on the structural order parameter. First, we will now briefly outline some general considerations how to treat such problem and then utilize, in addition, the model introduced in [20,21] to arrive at definite quantitative results.

Generally, any thermodynamic quantity, φ, describing the state of a liquid undergoing crystallization processes depends on temperature, *T*, pressure, *p*, and composition, $\left\{x_{i}\right\}$, provided the system is in a (metastable) equilibrium state. Here by $\left\{x_{i}\right\}$ the set of molar fractions of the different components in the liquid is denoted. For systems in non-equilibrium states, a dependence on an appropriate set of structural order parameters, $\left\{\xi_{j}\right\}$, has to be accounted for, in addition. In the present paper, we restrict the analysis to cases when one structural order parameter is sufficient for the description. We express this dependence as $\varphi =\varphi (T,p,\left\{x_{i}\right\};\tilde{\xi })$, where $\tilde{\xi }$ is defined as

(10) $$\tilde{\xi }=\frac{\left(\xi -\xi_{e}\right)}{\xi_{e}}.$$

By a truncated Taylor expansion of $\varphi =\varphi (T,p,\left\{x_{i}\right\};\tilde{\xi })$ with respect $\tilde{\xi }$ we obtain

(11) $$\varphi \left(T,p,\left\{x_{i}\right\};\tilde{\xi }\right)≅\varphi_{e}\left(T,p,\left\{x_{i}\right\}\right)+A_{\varphi }\tilde{\xi }+B_{\varphi }{\tilde{\xi }}^{2}+\ldots$$

The first term in the right hand side of Equation (11) supplies us with the value of φ in thermodynamic equilibrium ($\varphi =\varphi_{e}\left(T,p,\left\{x_{i}\right\}\right)$), the additional terms account for changes of this quantity due to deviations from equilibrium, they are equal to zero for $\xi =\xi_{e}$ or $\tilde{\xi }=0$.

Such approach can be employed also for the specification of the dependence of the relaxation time on the structural order parameter considering $U_{a}^{*}$ as a function of $\tilde{\xi }$. In a first-order approximation, we obtain

(12) $$U_{a}^{*}\left(T,p,\left\{x_{i}\right\};\tilde{\xi }\right)≅A_{0}\left(T,p,\left\{x_{i}\right\}\right)+A_{u}\left(T,p,\left\{x_{i}\right\}\right)\tilde{\xi },\;A_{u}\left(T,p,\left\{x_{i}\right\}\right)<0.$$

The inequality $A_{u}<0$ is a consequence of the fact that the viscosity of systems in non-equilibrium is, on cooling, smaller than the respective equilibrium viscosity [20,21,26,37,71,75,76,77,78]. Here we will restrict the considerations to the specification of the difference, Δμ(T,p;ξ), in the chemical potential per particle in the liquid and the crystal and the surface tension, σ(T,p;ξ).

The change of the Gibbs free energy in cluster formation, we will express approximately as [11,13,20,21,82]

(13) $$\Delta G\left(n\right)≅-n\Delta \mu \left(T,p;\xi \right)+\gamma \left(T,p;\xi \right)n^{2/3},$$

utilizing the notations

(14) $$\Delta \mu =d_{0}^{3}\Delta g,\;\;\gamma \left(T,p;\xi \right)=\Omega d_{0}^{2}\sigma \left(T,p;\xi \right),\;\Omega =4\pi {\left(\frac{3}{4\pi }\right)}^{2/3}.$$

Here $d_{0}$ is a characteristic size parameter determined by the particle number density ($c=(1/d_{0}^{3})$). Having at our disposal the values of Δμ(T,p;ξ) and σ(T,p;ξ), we can compute via Equation (13) the work of formation of a cluster containing *n* particles. In the present paper, we omit self-consistency corrections [13] to concentrate the attention onto the main topic of the present analysis.

Assuming that the liquid has reached the appropriate metastable equilibrium state prior to the initiation of nucleation (the condition $\xi =\xi_{e}$ is fulfilled), the thermodynamic driving force of crystal nucleation and the surface tension can be expressed as [9,11,12,13,82].

(15) $$\Delta \mu \left(T,p;\xi =\xi_{e}\right)≅\Delta h_{m}\left(1-\frac{T}{T_{m}}\right)\left[1-\frac{\Delta c_{p}}{2\Delta s_{m}}\left(1-\frac{T}{T_{m}}\right)\right],$$

(16) $$\frac{\sigma (T,p;\xi =\xi_{e})}{\sigma (T_{m},p_{m})}≅\left(\frac{T}{T_{m}}\right)\left[1-\frac{\Delta c_{p}}{\Delta s_{m}}\left(1-\frac{T}{T_{m}}\right)\right].$$

Here $\Delta h_{m}$ and $\Delta s_{m}$ are the melting enthalpy and melting entropy per particle of the crystal at the temperature, $T_{m}$, and the pressure, $p_{m}$, $\Delta c_{p}$ is the difference in specific heats per particle in the liquid and the crystalline phases, respectively, also at ($T_{m},p_{m}$). As the new element in the present analysis, we account now in addition for the possibility that the liquid is not in a state of metastable equilibrium.

At fixed temperature and pressure, a spontaneous evolution of any thermodynamic system is connected with a decrease of the Gibbs free energy [83,84]. For the problem under consideration, such processes can be illustrated by the motion of a ball in a force-field as shown in Figure 4a. The crystalline state corresponds to the lowest value of the Gibbs free energy as illustrated in Figure 4c. Relaxation processes result consequently in a decrease of the thermodynamic driving force of crystallization.

In the considered here model, the differences between the Gibbs free energy per particle are due to differences in the configurational contributions to this thermodynamic function. As shown in [67] and briefly explained in the Appendix A, the thermodynamic driving force of crystallization per particle of the liquid accounting for deviations from metastable equilibrium gets the form

(17) $$\Delta \mu \left(T,p;\xi \right)≅\Delta h_{m}\left(1-\frac{T}{T_{m}}\right)\left[1-\frac{\Delta c_{p}\left(T_{m},p_{m}\right)}{2\Delta s_{m}}\left(1-\frac{T}{T_{m}}\right)\right]+\frac{k_{B}T\xi_{e}}{2}{\tilde{\xi }}^{2},$$

i.e., the changes in the thermodynamic driving force of crystallization caused by deviations from metastable equilibrium are given by $\left(k_{B}T\xi_{e}/2\right){\tilde{\xi }}^{2}$.

On cooling, deviations of the state of the liquid from metastable equilibrium lead to higher values of the configurational entropy as compared with its metastable equilibrium state [20,21,49,50]. In line with our approach to the determination of the surface tension via entropy differences [9,11,12,13,82],

(18) $$\frac{\sigma (T,p,\xi )}{\sigma (T_{m},p_{m})}=\frac{T\Delta s(T,p;\xi )}{T_{m}\Delta s\left(T_{m},p_{m}\right)}=\frac{T}{T_{m}}\left(\frac{\Delta s\left(T,p;\xi_{e}\right)+s_{conf}\left(\xi \right)-s_{conf}\left(\xi_{e}\right)}{\Delta s(T_{m},p_{m})}\right),$$

deviations from equilibrium result generally in higher values of the surface tension. In mentioned approach, in line with the Stefan–Skapski–Turnbull relation, we express the surface tension via the enthalpy or entropy of melting. Consequently, in order to account for changes of the surface tension caused by different factors affecting it, one has to specify their effect on the melting entropy. Here, by $s_{conf}$, the configurational contribution to the entropy per particle in the liquid is denoted. For the particular model employed, we obtain the following correction term accounting for deviations of the liquid from metastable equilibrium

(19) $$\frac{\sigma (T,p,\xi )}{\sigma (T_{m},p_{m})}=\frac{T}{T_{m}}\left(1-\frac{\Delta c_{p}}{\Delta s_{m}}\left(1-\frac{T}{T_{m}}\right)+\frac{s_{conf}\left(\xi \right)-s_{conf}\left(\xi_{e}\right)}{\Delta s_{m}}\right)$$

or

(20) $$\frac{\sigma (T,p,\xi )}{\sigma (T_{m},p_{m})}=\frac{T}{T_{m}}\left[1-\frac{\Delta c_{p}}{\Delta s_{m}}\left(1-\frac{T}{T_{m}}\right)-\left(\frac{k_{B}\xi_{e}\ln\xi_{e}}{\Delta s_{m}}\right)\tilde{\xi }\right].$$

Consequently, both in the general case and in the particular realization utilizing the lattice-hole model discussed here so far, on cooling processes, deviations of the state of the liquid from equilibrium result in both an increase of the thermodynamic driving force and in an increase of the surface tension monotonically increasing with increasing degree of deviations from metastable equilibrium. Isothermal relaxation leads to a decrease of $\tilde{\xi }$ and results, consequently, in a decrease of both the thermodynamic driving force and the surface tension.

For heating processes, the situation remains the same with respect to the thermodynamic driving force (it is larger as compared to the state of metastable equilibrium of the liquid). However, deviations from equilibrium may result in a decrease of the surface tension as far as the condition $\tilde{\xi }<0$ is fulfilled. This reduction of the surface tension may eventually result in an increase of the intensity of crystal nucleation, as discussed in detail in [78]. Such effects are expected to be of significant importance also for the correct description of cold crystallization widely discussed in polymer physics [45]. However, here we concentrate the attention to crystallization processes proceeding after or in the course of cooling a liquid leaving mentioned problem to a future analysis.

In the present study, we will utilize these general consequences and particular results accounting also for a possibly much more complex behavior of real systems as compared to the features described by the simple lattice-hole model discussed so far. On one hand, we could utilize an approach as described with Equation (11) not involving any particular model. It leads to the following expressions for the thermodynamic driving force

(21) $$\Delta \mu \left(T,p;\xi \right)≅\Delta h_{m}\left(1-\frac{T}{T_{m}}\right)\left[1-\frac{\Delta c_{p}\left(T_{m},p_{m}\right)}{2\Delta s_{m}}\left(1-\frac{T}{T_{m}}\right)+{\tilde{\Omega }}_{\Delta \mu }{\tilde{\xi }}^{2}\right]$$

and the surface tension

(22) $$\frac{\sigma (T,p,\xi )}{\sigma (T_{m},p_{m})}=\frac{T}{T_{m}}\left[1-\frac{\Delta c_{p}}{\Delta s_{m}}\left(1-\frac{T}{T_{m}}\right)+{\tilde{\Omega }}_{\sigma }\tilde{\xi }\right].$$

Estimates show that, employing values of the parameters ${\tilde{\Omega }}_{\Delta \mu }$ and ${\tilde{\Omega }}_{\sigma }$ of the order of one, already significant contributions to the thermodynamic driving force and the surface tension caused by deviations of the state of the liquid, respectively, the glass from metastable equilibrium are obtained.

However, here we will use the following relations for these dependencies being straightforward generalizations of Equations (17) and (19) obtained in terms of the simple lattice-hole model described in the Appendix A:

(23) $$\Delta \mu \left(T,p;\xi \right)≅\Delta h_{m}\left(1-\frac{T}{T_{m}}\right)\left[1-\frac{\Delta c_{p}\left(T_{m},p_{m}\right)}{2\Delta s_{m}}\left(1-\frac{T}{T_{m}}\right)\right]+\Omega_{\Delta \mu }\left(\frac{k_{B}T\xi_{e}}{2}\right){\tilde{\xi }}^{2},$$

(24) $$\frac{\sigma (T,p,\xi )}{\sigma (T_{m},p_{m})}=\frac{T}{T_{m}}\left[1-\frac{\Delta c_{p}}{\Delta s_{m}}\left(1-\frac{T}{T_{m}}\right)-\Omega_{\sigma }\left(\frac{k_{B}\xi_{e}\ln\xi_{e}}{\Delta s_{m}}\right)\tilde{\xi }\right].$$

Note that, since the inequalities $0<\xi_{e}<1$ and $\ln\xi_{e}<0$ hold, deviations from metastable equilibrium result in an increase of the surface tension as it should be generally the case. Qualitatively, both models given by Equations (21) and (22), respectively, Equations (23) and (24), lead to similar results.

**Figure 5:**
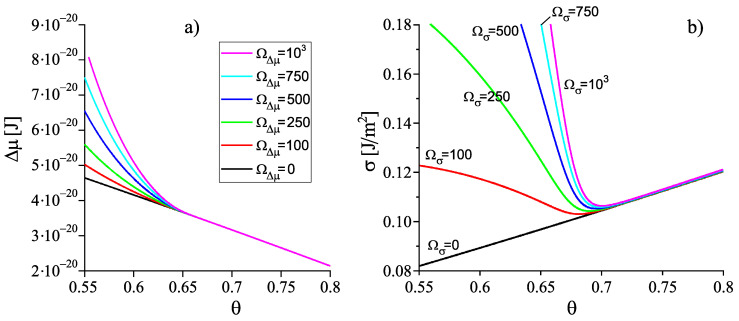
Determination of the dependence of (**a**) the thermodynamic driving force and (**b**) the surface tension on reduced temperature, $\theta =T/T_{m}$, for the cases when the liquid deviates on cooling from metastable equilibrium in the form as illustrated in Figure 3. The curves are drawn based on Equations (23) and (24) with values of the parameters equal to $\Omega_{\Delta \mu }=\Omega_{\sigma }=0,100,250,500,750,1000$. Simultaneously also the respective dependencies are given for the case that the liquid retains always in a state of metastable equilibrium ($\tilde{\xi }=0$ or $\Omega_{\Delta \mu }=\Omega_{\sigma }=0$). Parameters are taken widely from our recent paper [13]: $T_{m}=1307$ K, $\Delta H_{m}=9.744\times 10^{8}$ J/m^3^, and $\Delta c_{p}/\Delta s_{m}=0.275$, $d_{0}=4.8\times 10^{-10}$ m, $\sigma_{\infty }=0.170\;J/m^{2}$.

The results of computations of the dependence of the thermodynamic driving force and the surface tension on reduced temperature, $\theta =T/T_{m}$, based on Equations (23) and (24) are shown in Figure 5. In the computations it is assumed, again, that the liquid deviates on cooling from metastable equilibrium in the form as illustrated in Figure 3. Only for systems where the value of the structural order parameter may affect the thermodynamic driving force and the surface tension significantly, deviations of the liquid from metastable equilibrium may be of relevance for nucleation and growth in crystallization. Consequently, we will assign such values to the parameters $\Omega_{\Delta \mu }$ and $\Omega_{\sigma }$ that this necessary condition for the applicability of our model is fulfilled, i.e., such choice of the parameters is performed to correct limitations of the model employed in the description of structural relaxation processes.

**Figure 6:**
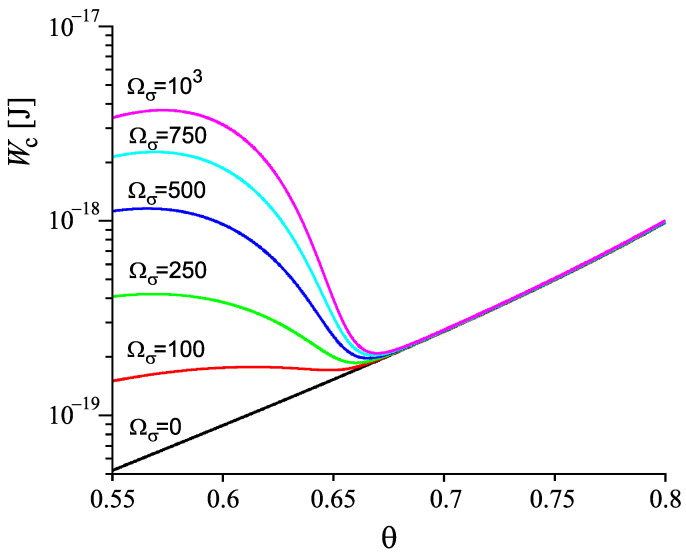
Determination of the dependence of the work of critical cluster formation on reduced temperature, $\theta =T/T_{m}$, for the cases when the liquid deviates on cooling from metastable equilibrium in the form as illustrated in Figure 3. The curves are drawn based on Equations (23)–(25) with values of the parameters equal to $\Omega_{\Delta \mu }=\Omega_{\sigma }=100,250,500,750,1000$, again. Simultaneously also the respective dependencies are given for the case that the liquid retains always in a state of metastable equilibrium ($\tilde{\xi }=0$ or $\Omega_{\Delta \mu }=\Omega_{\sigma }=0$). Again, the liquid deviates on cooling from metastable equilibrium in the form as illustrated in Figure 3. For the values of the other parameters, see caption to Figure 5.

Having at our disposal the expressions for the thermodynamic driving force of crystallization and the surface tension, we can compute the work of critical cluster formation in dependence on temperature via Equation (1) [8,9,13,20,21]. With cΔμ=Δg, we obtain from Equation (1)

(25) $$W_{c}\left(T,p;\xi \right)=\Delta G\left(n_{c}\right)=\frac{16\pi }{3}\frac{\sigma^{3}\left(T,p;\xi \right)}{{\left(c\Delta \mu \left(T,p;\xi \right)\right)}^{2}},\;c=\frac{1}{d_{0}^{3}},$$

where Δμ and σ are given by Equations (23) and (24). The dependence of $\tilde{\xi }$ on temperature is taken from the results of computations described here earlier and presented in Figure 3. The results of computations of $W_{c}$ are shown in Figure 6. In comparison to $W_{c}\left(T,p;\xi \right)$ and σ(T,p;ξ), the work of critical cluster formation is also presented for the case that on cooling the system is retained in the respective metastable equilibrium state of the liquid, i.e., for the case $W_{c}\left(T,p;\xi_{e}\right)$. A comparison with Figure 2 shows a complete qualitative agreement. The account of deviations of the state of the liquid respectively the glass from metastable equilibrium leads to a dependence of the work of critical cluster formation on temperature as it has to be the case to interpret correctly the experimental data in terms of CNT.

**Figure 7:**
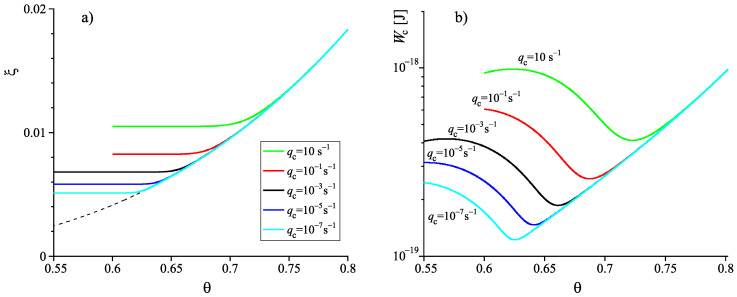
Dependence of (**a**) the structural order parameter, ξ, and (**b**) the work of critical cluster formation, $W_{c}$, on reduced temperature for different cooling rates, $q_{c}=\left|dT/dt\right|$ as specified in the figure. Here we assigned to the parameters Ω the following values $\Omega_{\Delta \mu }=\Omega_{\sigma }=250$.

Once the structural order parameter depends on cooling rate, also surface tension, thermodynamic driving force and work of critical cluster formation at some given temperature depend on the cooling rate the system is transferred to it. This feature is illustrated in Figure 7. It is shown that variations of the cooling rate may result in significant changes of the dependence of the work of critical cluster formation on temperature. It is evident that the curves are, again, qualitatively similar to the results given in Figure 2 showing the course of the work of critical cluster formation required for an interpretation of experimental data.

In experiments on crystal nucleation, the nucleation temperature may also be established in alternative ways. Consequently, the degree of deviation from metastable equilibrium reached initially at the chosen nucleation temperature may be different for different thermal histories. In modeling a particular experimental situation, one has to account therefore for how the initial state is established to obtain the correct initial values of the structural order parameter, and as its consequence, the thermodynamic driving force and the surface tension. The method of description of the effect of structural relaxation on the nucleation kinetics requires exclusively the knowledge of this initial value of the structural order parameter. Here we will retain the model of cooling with a constant rate down to the nucleation temperature for the specification of the initial value of the structural order parameter as described in Figure 3.

As the next step we now have to analyze at which conditions the results shown in Figure 6 and Figure 7 or reached via alternative ways to generate the initial state may significantly affect the nucleation kinetics.

## 4. Implications for the Description of Crystal Nucleation: Kinetic Aspects

### 4.1. Relaxation and Crystal Nucleation: Analytical Estimates

Whether or not or to which extent the considerations outlined in Section 3 will be of relevance for the description of crystal nucleation depends on the answer to the question, what the characteristic time-scales of crystal nucleation are in comparison with the characteristic times of relaxation of the liquid to the metastable equilibrium state. Here we would like to demonstrate first, why in CNT it is so far generally assumed that relaxation is practically completed prior to nucleation.

To answer this question one has to define first a characteristic time-scale of crystal nucleation. As such a time-scale, we will employ (as it is conventionally done [20,21,41,42,85,86,87,88]) the average time, 〈τ〉, of formation of the first supercritical crystal nucleus. As shown recently in [89], the average time of formation of the first supercritical nucleus can be expressed in a good approximation as

(26) $$\langle \tau \rangle ≅\tau_{ns}+{\langle \tau \rangle }_{ss},\;\;{\langle \tau \rangle }_{ss}=\frac{1}{J_{st}V}.$$

Here $\tau_{ns}$ is the time-lag in nucleation, the characteristic time required to establish a steady-state cluster size distribution up to clusters of critical sizes [20,21,90]. Provided steady-state conditions with respect to nucleation are established, the following relation

(27) $$\langle \tau \rangle ={\langle \tau \rangle }_{ss}=\frac{1}{J_{st}V},$$

holds for the time required to form the first supercritical cluster at such conditions. Here $J_{st}\left(T\right)$ is the steady-state nucleation rate per unit volume and *V* is the volume of the ambient phase, in our case, the liquid.

The time-lag and the steady-state nucleation rate in application to crystal nucleation can be expressed as [13,20,21]

(28) $$\tau_{ns}=\frac{2\omega \sigma k_{B}T}{{\left(d_{0}\Delta g\right)}^{2}}\left(\frac{1}{D}\right)=\frac{\omega }{2}\left(\frac{k_{B}T}{\sigma d_{0}^{2}}\right)\left(\frac{R_{c}^{2}}{D}\right),$$

(29) $$J_{st}=c\sqrt{\frac{\sigma }{k_{B}T}}\left(\frac{2D}{d_{0}}\right)\exp\left(-\frac{W_{c}}{k_{B}T}\right).$$

Here *D* is the diffusion coefficient governing the aggregation kinetics, the numerical factor ω varies in the range 1≤ω≤4 depending on the method employed in the derivation of Equation (28). According to Equation (26), both quantities, $\tau_{ns}$ and ${\langle \tau \rangle }_{ss}$, contribute to the average time of formation of the first supercritical nucleus, 〈τ〉. However, as demonstrated in [89], their contributions are quite different in different temperature ranges.

To prove this statement, we have a brief look at the ratio $\left(\tau_{ns}/{\langle \tau \rangle }_{ss}\right)$. Near to the melting temperature, it obeys the inequality

(30) $$\frac{\tau_{ns}}{{\langle \tau \rangle }_{ss}}=\tau_{ns}\left(J_{st}V\right)=\omega \sqrt{\frac{k_{B}T}{\sigma }}\left(\frac{R_{c}^{2}V}{d_{0}^{6}}\right)\exp\left(-\frac{W_{c}}{k_{B}T}\right)\ll 1\;\mathrm{at}\;T\to T_{m}.$$

Both the critical cluster radius, $R_{c}$, and the work of critical cluster formation, $W_{c}$, diverge at the approach to $T_{m}$, however, the exponential term dominates resulting in $\tau_{ns}/{\langle \tau \rangle }_{ss}\to 0$ at $T\to T_{m}$. This dominance is extended down to temperatures near but sufficiently away from temperatures, $T_{max}$, corresponding to the maximum of the steady-state nucleation rate. For lower temperatures, the inequality is reversed, i.e., $\left(\tau_{ns}/{\langle \tau \rangle }_{ss}\right)$ becomes considerably larger than one [48,49,50]. We arrive at the conclusion that, in the range of temperatures of interest for us ($T⪅1.2T_{g}^{\left(T\right)}$) near and below the glass transition temperature, the average time of formation of the first supercritical nucleus is determined mainly by the time-lag in nucleation,

(31) $$\langle \tau \rangle ≅\tau_{ns}=\frac{\omega }{2}\left(\frac{k_{B}T}{\sigma d_{0}^{2}}\right)\left(\frac{R_{c}^{2}}{D}\right)\;\mathrm{at}\;T⪅1.2T_{g}^{\left(T\right)}.$$

Consequently, we have finally to analyze how time-lag and relaxation time are related in the considered range of temperatures.

To arrive at the respective estimates, in CNT commonly two additional assumptions are introduced. First, the Stokes–Einstein–Eyring equation [20,21]

(32) $$D≅\frac{k_{B}T}{d_{0}\eta }$$

is utilized, correlating diffusion coefficient, *D*, and Newtonian viscosity, η. As a second step, the relaxation time can be expressed by the viscosity via the Maxwell relation [20,21,45,91,92]

(33) $$\tau_{R}=\frac{\eta }{G^{*}},\;G^{*}=\frac{E}{2(1+\gamma )}.$$

Here $G^{*}$ is the infinite frequency shear modulus, *E* is Young’s modulus and γ is the Poisson number. The relation between viscosity and relaxation time can be written also in the form [20,21]

(34) $$\tau_{R}≅\frac{d_{0}^{3}}{k_{B}T}\eta .$$

In the present model analysis, we will utilize latter result. A combination of Equations (31), (32) and (34) results in

(35) $$\langle \tau \rangle ≅\tau_{ns}=\frac{\omega }{2}\left(\frac{k_{B}T}{\sigma d_{0}^{4}}\right)R_{c}^{2}\tau_{R}\propto \left(\frac{k_{B}T}{\sigma d_{0}^{2}}\right)n_{c}^{2/3}\tau_{R}\;\mathrm{at}\;T⪅1.2T_{g}^{\left(T\right)}.$$

Employing, finally, the capillarity approximation in the description of experimental data on steady-state nucleation rates, typical values of the term $\left(k_{B}T/\sigma d_{0}^{2}\right)$ in Equation (35) are found in the range of $10^{2}$–$10^{3}$ [20,21,42]. Provided these estimates and the underlying them assumptions are true, then the characteristic times of nucleation, $\langle \tau \rangle ≅\tau_{ns}$, are always much larger as compared to the times of relaxation, $\tau_{R}$, to the metastable equilibrium state of the liquid, i.e., nucleation proceeds only after the liquid is transferred into the respective metastable state. However, is this conclusion really always correct?

As discussed in detail in [13,19,20,21,30,93], the interpretation of experimental data on steady-state nucleation rates utilizing the capillarity approximation results as a rule in serious problems. In particular, to the size parameter, $d_{0}$, values have to be assigned to which do not correspond to their original meaning, the values are too small. This is one of the reasons for the high values in the estimates of the parameter $\left(k_{B}T/\sigma d_{0}^{2}\right)$ in Equation (35). The introduction of a curvature dependence of the surface tension partly resolves these problems and results in estimates of the order $\langle \tau \rangle ≅\tau_{ns}≅\tau_{R}$ near to the maximum of the steady-state nucleation rate. Further, one can also advance theoretical arguments leading to the conclusion that sufficiently below $T_{g}^{\left(T\right)}$ the inequality $\tau_{ns}\ll \tau_{R}$ should hold as a rule [26,48,49,50]. We will return here to this topic in Section 5.

Another serious argument against the conclusions derived from Equation (35) (relaxation proceeds always prior to nucleation) is connected with another assumption involved in its derivation. It consists in the application of the Stokes–Einstein–Eyring relation in the description of crystal nucleation. It is well-known that this relation is as a rule fulfilled only above a certain decoupling temperature, $T_{d}$ [94], but not in the range $T\leq T_{d}≅\left(1.1-1.2\right)T_{g}^{\left(T\right)}$. In the model computations, we will set $T_{d}=1.2T_{g}^{\left(T\right)}$. In such case, based on Equation (31), we obtain

(36) $$\langle \tau \rangle ≅\tau_{ns}=\frac{\omega }{2}\left(\frac{k_{B}T}{\sigma d_{0}^{4}}\right)\left(\frac{R_{c}^{2}d_{0}^{2}}{D\tau_{R}}\right)\tau_{R}\;\mathrm{at}\;T⪅T_{d}≅1.2T_{g}^{\left(T\right)}$$

instead of Equation (35). Consequently, in the general relation, Equation (36), not $R_{c}^{2}$ (as in Equation (35)) but $R_{c}^{2}d_{0}^{2}/\left(D\tau_{R}\right)$ occurs as a factor for the determination of the average time of formation of the first critical nucleus at otherwise identical conditions. Consequently, this relation, Equation (36), will lead, in general, to quite different predictions as compared with Equation (35) and the conclusions derived based on Equation (35) are shown in this way to be, in general, not correct.

In particular, in [95] effects of decoupling of diffusion and viscosity on crystal growth have been analyzed for a variety of liquids, including those presented in Figure 1. The temperature dependence of viscosity is described in a wide temperature range with good accuracy by the Vogel–Fulcher–Tammann (VFT) equation,

(37) $$\eta =\eta_{0}\exp\left(\frac{E_{*}}{k_{B}\left(T-T_{0}\right)}\right).$$

To account appropriately for decoupling of diffusion and viscosity, a simple model was employed adapting a suggestion formulated by Rössler [96]. The diffusion coefficient was supposed to behave differently above and below the decoupling temperature $T_{d}$ as

(38) $$D=\left\{\begin{array}{cc} D_{0}\exp\left(-\frac{E_{*}}{k_{B}\left(T-T_{0}\right)}\right) & \mathrm{for}\;T\geq T_{d}\\ \; & \\ D_{0}\exp\left(-\frac{E_{*}}{k_{B}T\left(1-\frac{T_{0}}{T_{d}}\right)}\right) & \mathrm{for}\;T\leq T_{d} \end{array}\right.$$

Here it is accounted for that the mechanism controlling crystal growth changes from a liquid-like mode, correlated with the dynamics of relatively large cooperatively rearranging regions, into a solid-state-like (faster) diffusion mode governed by an Arrhenius-type process. In the latter mode, the activation energy for diffusion does not depend on temperature [97] (see also [98,99,100,101]). With such an assumption, the time-lag in nucleation has to be expected according to Equation (28) to also exhibit a temperature dependence of Arrhenius type. This expectation is confirmed by experimental data on the time-lag in crystal nucleation discussed in [19]. Equation (38) we will utilize in the present model analysis of the effect of relaxation of the liquid on crystal nucleation. The implementation of these ideas into the model analysis will be discussed in the next section.

### 4.2. Relaxation and Crystal Nucleation: Numerical Computations

We model crystal nucleation and growth processes by the standard set of kinetic equations employed in CNT [20,21] in the form as described in [13] and their analytical consequences. Kinetic aspects enter CNT by the appropriate diffusion coefficient, *D*, or the Newtonian viscosity, η, of the liquid. As discussed in detail in [13,20,21], the attachment coefficients can be written in the form

(39) $$w^{\left(+\right)}\left(n,t\right)=\Omega \frac{D}{d_{0}^{2}}n^{2/3},\;w^{\left(+\right)}\left(n,t\right)=\Omega \frac{k_{B}T}{d_{0}^{3}\eta }n^{2/3}.$$

Consequently, as the first of the tasks in the analysis of the effects of deviations of the liquid from metastable equilibrium and relaxation on crystallization, we have to formulate the prescription how we are going to express the diffusion coefficient of the particles in the liquid for the model considered.

As already noted in Section 2, we describe the dependence of relaxation time on temperature via Equations (7)–(9). A combination of Equations (9) and (34) yields the following expression for the viscosity

(40) $$\eta =\frac{k_{B}T}{d_{0}^{3}}\tau_{R}=\frac{k_{B}T}{d_{0}^{3}}\tau_{0}\exp\left(7.5\frac{T_{g}^{\left(T\right)}}{T-T_{0}}\right).$$

For temperatures above the decoupling temperature, $T_{d}$, the Stokes–Einstein–Eyring relation, Equation (34), holds. Using it, with Equation (40) we can determine the diffusion coefficient in this temperature range. The pre-exponential term $D_{0}$ in Equation (38) is obtained then as $D_{0}=\left(d_{0}^{2}/\tau_{0}\right)$ and the activation energy for viscous flow as $\left(E_{*}/k_{B}\right)=7.5T_{g}^{\left(T\right)}$. Employing these replacements also for temperatures below the decoupling temperature, Equation (38) leads to the following expression for the diffusion coefficient

(41) $$D=\left\{\begin{array}{cc} \left(\frac{d_{0}^{2}}{\tau_{0}}\right)\exp\left(-7.5\frac{T_{g}^{\left(T\right)}}{T-T_{0}}\right) & \mathrm{for}\;T\geq T_{d}\\ \; & \\ \left(\frac{d_{0}^{2}}{\tau_{0}}\right)\exp\left(-7.5\frac{T_{g}^{\left(T\right)}}{T}\left(\frac{T_{d}}{T_{d}-T_{0}}\right)\right) & \mathrm{for}\;T\leq T_{d} \end{array}\right.$$

With these relations, we obtain from Equation (36)

(42) $$\langle \tau \rangle ≅\tau_{ns},\;\;\frac{\tau_{ns}}{\tau_{R}}=\frac{\omega }{2}\left(\frac{k_{B}T}{\sigma d_{0}^{2}}\right)\left(\frac{R_{c}^{2}}{d_{0}^{2}}\right)\exp\left(-\frac{7.5T_{g}^{\left(T\right)}T_{0}\left(T_{d}-T\right)}{T\left(T-T_{0}\right)\left(T_{d}-T_{0}\right)}\right)$$

$$T⪅T_{d}≅1.2T_{g}^{\left(T\right)}$$

**Figure 8:**
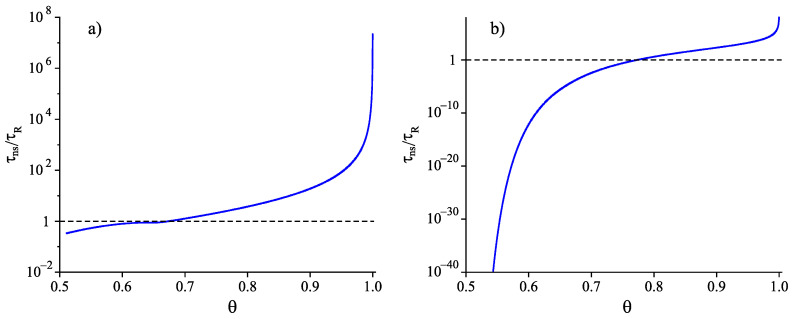
Dependence of the ratio of time-lag in nucleation and Maxwellian relaxation time, $\tau_{ns}/\tau_{R}$, on the reduced temperature for the model system under consideration if (**a**) the Stokes–Einstein–Eyring equation is utilized (Equation (35)), and (**b**) in the general and more correct approach that decoupling of diffusion and relaxation is accounted for (Equation (42)). As earlier, $\Omega_{\Delta \mu }=\Omega_{\sigma }=250$.

In comparison with Equation (35) involving in its derivation the Stokes–Einstein–Eyring equation, an exponential term occurs in Equation (42). Being equal to one at $T=T_{d}$, this term decreases significantly and monotonically with a decrease of temperature becoming equal to zero at $T=T_{0}$. It follows that the characteristic time of nucleation may become much shorter in this range of temperatures than the relaxation time. This conclusion is illustrated in Figure 8. Such behavior is not found if the Stokes–Einstein–Eyring equation is utilized in the theoretical description.

**Figure 9:**
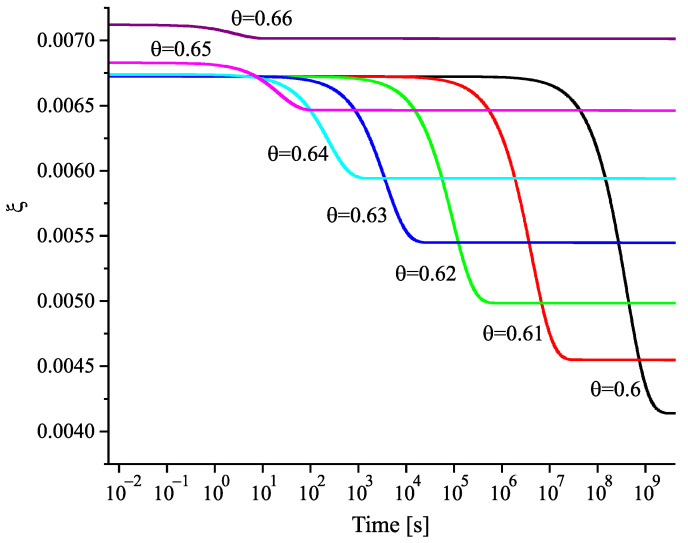
Dependence of the structural order parameter on time for different values of the reduced temperature, $\theta =T/T_{m}$. The evolution in time is caused by isothermal relaxation of the liquid to the metastable equilibrium state described by Equation (4).

**Figure 10:**
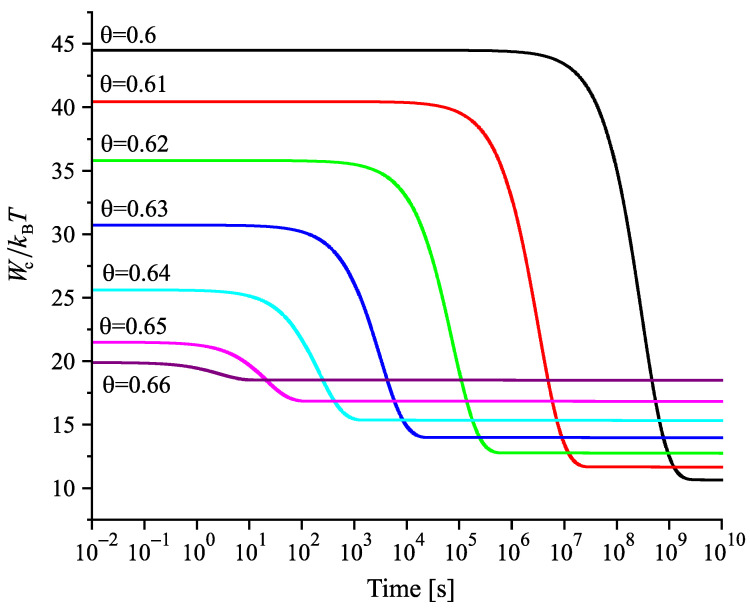
Time dependence of the work of critical cluster formation caused by isothermal relaxation of the liquid described by the time evolution of the structural order parameter as illustrated in Figure 9 for reduced temperatures θ=0.6,0.61,0.62,0.62,0.64,0.65,0.66. The evolution starts with a value of ξ obtained on cooling with a rate of change of temperature as described in Figure 3 (see text).

**Figure 11:**
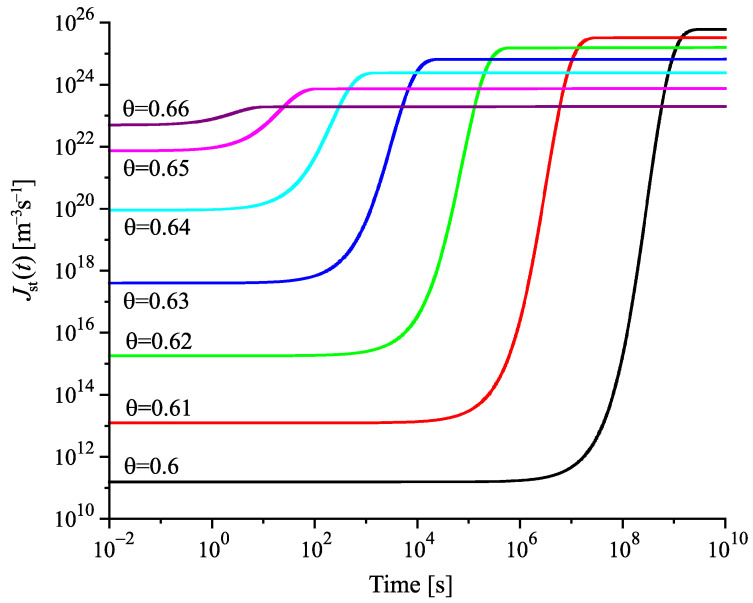
Time dependence of the steady-state nucleation rate caused by isothermal relaxation of the liquid described by the structural order parameter as illustrated in Figure 9 and the work of critical cluster formation illustrated in Figure 10 for reduced temperatures θ=0.6,0.61,0.62,0.62,0.64,0.65,0.66. The evolution starts with a value of ξ obtained on cooling with a rate of change of temperature as described in Figure 3 (see text).

**Figure 12:**
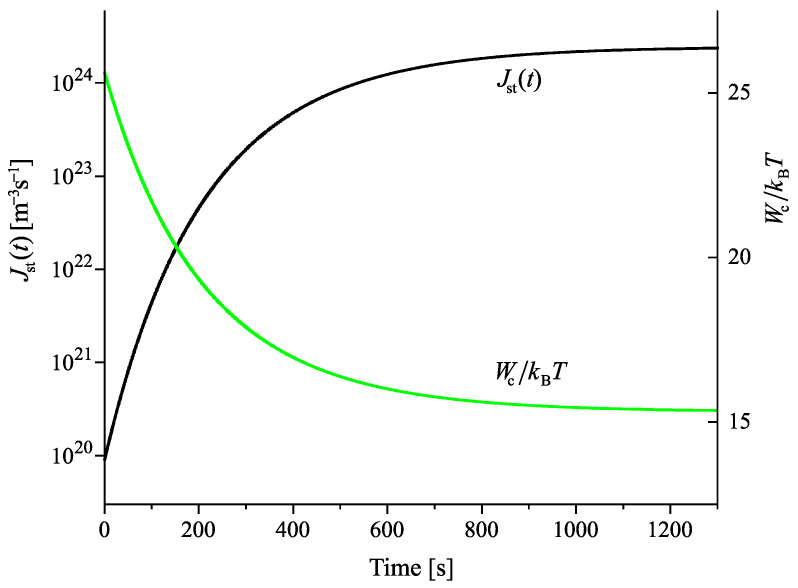
Time dependence of the work of critical cluster formation and the steady-state nucleation rate caused by isothermal relaxation of the liquid described by the structural order parameter for a reduced temperature equal to θ=0.64 (see text).

Having at our disposal the tools for the analysis, we can get the desired information on the nucleation behavior by solving the respective kinetic equations or utilizing the analytical expressions, Equations (25) and (29). Hereby, we always start at the melting temperature and cool the system with a given rate down to a specified temperature near or below $T_{g}^{\left(T\right)}$. Reaching the value of the chosen temperature, we follow the development of the nucleation rate. We suppose that initially a value of the structural order parameter is reached as described in Figure 3. Isothermal relaxation of the structural order parameter towards its equilibrium value results in changes of the work of critical cluster formation and the steady-state nucleation rate. The results are shown for different values of reduced temperature in Figure 9, Figure 10 and Figure 11. For a relatively high value of temperature in the glass transition range (θ=0.64), the time evolution of the work of critical cluster formation and the steady-state nucleation rate are shown in Figure 12.

The behavior illustrated with Figure 9, Figure 10, Figure 11 and Figure 12 can be interpreted analytically. According to Equations (4) and (10), the change of the structural order parameter caused by isothermal relaxation processes is given by

(43) $$\frac{d\tilde{\xi }}{dt}=-\frac{\tilde{\xi }}{\tau_{R}\left(T,p,\xi \right)},\;\;\tilde{\xi }=\frac{\left(\xi -\xi_{e}\right)}{\xi_{e}}.$$

As shown in [37,71], an account of the dependence of the relaxation time on the structural order parameter results in stretched exponential (Kohlrausch or Jenckel) relations for the description of the relaxation behavior. Here we assume that the relaxation time depends only on pressure and temperature kept constant in the experiment analyzing nucleation. Consequently, Equation (43) then yields

(44) $$\tilde{\xi }\left(\tilde{t}\right)=\tilde{\xi }\left(0\right)\exp\left(-\tilde{t}\right),\;\tilde{t}=\frac{t}{\tau_{R}\left(T,p\right)}.$$

Employing this result, one obtains the dependencies for the work of critical cluster formation and the steady-state nucleation rate as shown in the figures.

For a comparison of the behavior of the structural order parameter at different temperatures, we introduce in addition the quantity

(45) $${\tilde{\xi }}_{m}=\frac{\xi \left(0\right)-\xi \left(\tilde{t}\right)}{\xi \left(0\right)-\xi_{e}}=\frac{1}{\xi \left(0\right)-\xi_{e}}\left[\left(\xi \left(0\right)-\xi_{e}\right)-\left(\xi \left(\tilde{t}\right)-\xi_{e}\right)\right]=1-\exp\left(-\tilde{t}\right).$$

**Figure 13:**
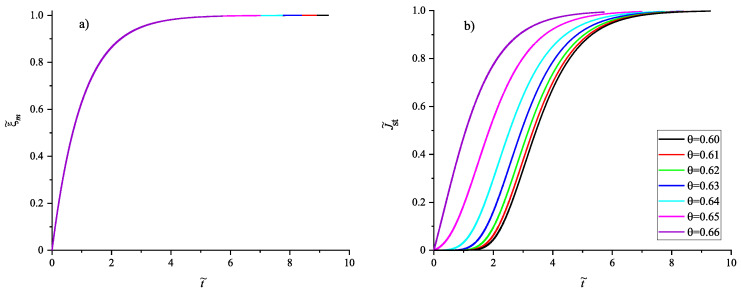
Change of (**a**) the modified structural order parameter (defined by Equation (45)) and (**b**) the steady-state nucleation rate in reduced form (Equation (46)) at isothermal annealing in dependence on the reduced time, $\tilde{t}=\left(t/\tau_{R}\right)$.

This quantity varies always from zero to one. Its time dependence is shown in Figure 13a, in line with Equation (45) its course does not depend on temperature. In similarly reduced units

(46) $${\tilde{J}}_{st}\left(t\right)=\frac{J_{st}\left(\xi \left(t\right)\right)-J_{st}\left(\xi \left(t=0\right)\right)}{J_{st}\left(\xi_{e}\right)-J_{st}\left(\xi \left(t=0\right)\right)},$$

the change of the steady-state nucleation rate in isothermal annealing is shown in Figure 13b in dependence on the reduced time, $\tilde{t}=\left(t/\tau_{R}\left(\theta \right)\right)$. ξ(t=0) is the initial value of the structural order parameter reached on cooling by the chosen rate and $\xi_{e}$ is the value at metastable equilibrium of the liquid. As evident, the curves obtained for ${\tilde{J}}_{st}\left(\tilde{t}\right)$ shown in Figure 13b coincide not completely but widely. The characteristic time to reach the ultimate value of the steady-state nucleation rate is determined, consequently, by the structural relaxation time.

It follows that the characteristic times of approach of the ultimate time-independent steady-state nucleation rates near and below the glass transition temperature are determined for all temperatures studied by the Maxwellian relaxation time. In line with the results shown in Figure 8, the relaxation time is much larger in this temperature ranges near and below $T_{g}^{\left(T\right)}$ as compared to the time-lag in nucleation. Consequently, we have here a situation quite different to the standard situation at isothermal crystallization widely analyzed in CNT, the approach to the ultimate steady-state conditions is not governed here by the time-lag in nucleation but by the relaxation time of the liquid to the metastable equilibrium state (cf. [39,40]). Note that this effect will be even more pronounced if relaxation is described more correctly by stretched exponential relaxation. The corresponding quantitative modifications will be studied in a future analysis, qualitatively, the results as outlined here will remain the same.

## 5. Account of Stress Evolution and Stress Relaxation in Crystal Nucleation

Crystallization of liquids or solids is accompanied by a change of the volume per unit mass or per particle. These deviations of the volume may result in the evolution of elastic stresses in crystal nucleation and growth in solids. Such stresses are of negligible effect in crystallization in liquids at sufficiently low viscosities. However, on cooling a liquid, in the vicinity of the glass-transition temperature and below, elastic stresses may evolve and have to be accounted for in the description of crystal nucleation [102,103] and crystal growth [104,105] processes.

A theory accounting for the interplay of stress development and stress relaxation on crystal nucleation has been developed in [20,21,22,23] and applied by us to the description of crystal nucleation in glass-forming liquids in [19,24,25,26]. In [25], it was shown that the account only of elastic stresses cannot explain to full extent the differences of theoretical results and experimental data on the steady-state nucleation rates as shown in Figure 1. However, stresses have to be accounted for and, by this reason, we will briefly analyze here in which way they either increase or decrease the effect of deviations of the liquid from the metastable equilibrium state on crystal nucleation. Moreover, we extend here the analysis of elastic stress effects in nucleation accounting for their influence on the surface tension of critical crystallites of the newly evolving phase.

Incorporating elastic stresses into the description of crystal nucleation, the change of the Gibbs free energy in cluster formation has to be written instead of Equation (13) in the form [13,20,21,22,23]

(47) $$\Delta G\left(n\right)≅-n\left(\Delta \mu \left(T,p;\xi \right)-\varepsilon \left(n\right)\right)+\gamma \left(T,p;\xi \right)n^{2/3}.$$

If nucleation proceeds in a Hookean solid (for that case we use the notation $\varepsilon =\varepsilon_{0}$), the parameter $\varepsilon =\varepsilon_{0}$ is determined by the elastic constants of both phases and (provided, as assumed here, that stresses are caused by density difference in both phases) does not depend on the number of particles in the crystallite. As shown in detail in [20,21,102], this parameter can become comparable in magnitude with Δμ.

For crystal nucleation in viscous liquids, the effective value of the stress parameter ε is determined by the interplay of stress evolution (caused by the formation of a crystallite) and stress relaxation accompanying this process. Assuming, as done here, that relaxation is described by Maxwell’s relaxation law, the effective value of ε for a crystallite of critical size is given by (see [20,21,102])

(48) $$\frac{\varepsilon (n_{c})}{\varepsilon_{0}}≅\frac{\tau_{R}}{\tau_{ns}}\left[1-\exp\left(-\frac{\tau_{ns}}{\tau_{R}}\right)\right].$$

**Figure 14:**
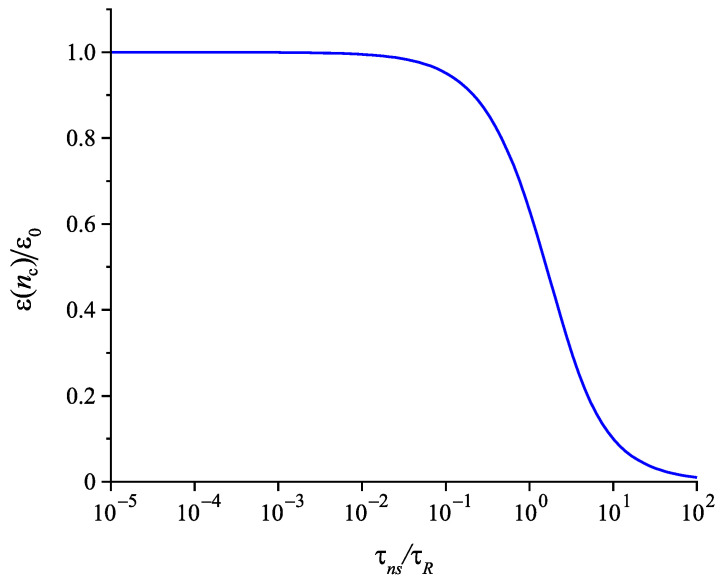
Dependence of the ratio $\left(\varepsilon \left(n_{c}\right)/\varepsilon_{0}\right)$ on the ratio $\tau_{ns}/\tau_{R}$ according to Equation (48). This ratio determines the decrease of the thermodynamic driving force in crystal nucleation caused by elastic stresses accounting for stress evolution and stress relaxation in highly viscous liquids.

**Figure 15:**
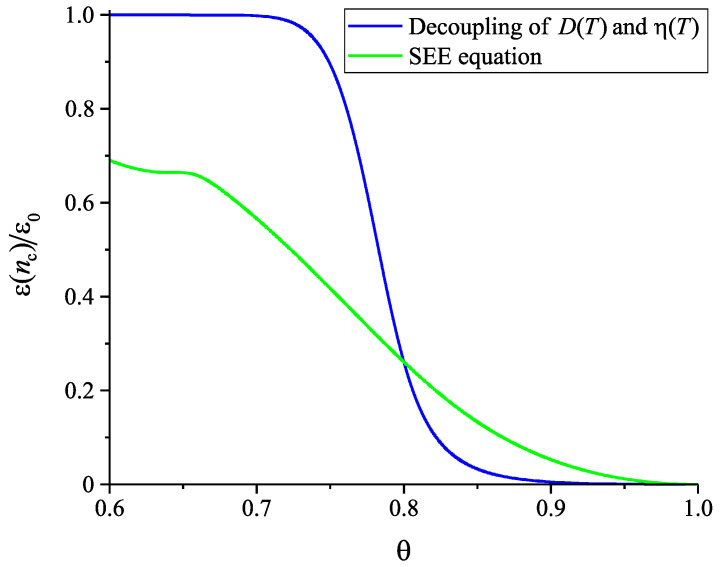
Dependence of the ratio $\left(\varepsilon \left(n_{c}\right)/\varepsilon_{0}\right)$ on temperature computed (green curve) via Equations (35) and (48) and (blue curve) via Equations (42) and (48). Only when decoupling of diffusion and relaxation is accounted for, a correct description of essential features of elastic stress effects on crystallization is incorporate in the theory, the transition from a viscous liquid at high to a Hookean solid at low temperatures.

Accounting for Equations (36) and (42), Equation (48) describes, at least, qualitatively correctly, the transition from nucleation in a liquid to nucleation in a solid as illustrated in Figure 14. In Figure 15, this ratio, $\left(\varepsilon \left(n_{c}\right)/\varepsilon_{0}\right)$, is shown in dependence on reduced temperature. For high temperatures near to the melting temperature, $T_{m}$, elastic stresses relax in the course of formation of a critical crystallite. For temperatures below the glass transition temperature, relaxation can be neglected and crystal nucleation proceeds as in a Hookean solid. An account of stretched exponential relaxation or a dependence of the relaxation time on the structural order parameter leads to an increase of $\varepsilon (n_{c})$ as compared with the predictions of Equation (48) [22,23].

Moreover, in line with cited previous studies, we use here the structural relaxation time for the description of stress relaxation. Of course, as discussed from a theoretical point of view in [92,104] and confirmed by experiment, the characteristic times of stress relaxation may differ from the structural relaxation times. Such modifications do not lead to qualitative changes of the main conclusion: Elastic stress effects may affect crystal nucleation only in cases when the structural relaxation time is considerably larger than the time-lag in nucleation, i.e., at conditions when crystallization proceeds concomitantly with structural relaxation. Their role in crystal nucleation increases with decreasing temperature.

Completing previous studies, finally, we would like to advance estimates now of the effect of elastic stresses on the surface tension. Melting a critical crystallite, the elastic energy generated by its evolution results in an additional contribution to the melting enthalpy. Utilizing the methods developed in [9,11,12,13,82] and applied here in the derivation of Equation (20), we may formulate similarly to Equation (18) the following relation to account for the dependence of the surface tension on elastic stresses

(49) $$\frac{\sigma (T,p,\xi ,\varepsilon )}{\sigma (T_{m},p_{m})}=\frac{T\Delta s(T,p;\xi )+\varepsilon }{T_{m}\Delta s\left(T_{m},p_{m}\right)},$$

resulting instead of Equation (20) in

(50) $$\frac{\sigma (T,p,\xi ,\varepsilon )}{\sigma (T_{m},p_{m})}=\frac{T}{T_{m}}\left[1-\frac{\Delta c_{p}}{\Delta s_{m}}\left(1-\frac{T}{T_{m}}\right)-\left(\frac{k_{B}\xi_{e}\ln\xi_{e}}{\Delta s_{m}}\right)\tilde{\xi }\right]+\frac{\varepsilon (n_{c})}{T_{m}\Delta s_{m}},$$

or, with Equation (48), in

(51) $$\frac{\sigma (T,p,\xi ,\varepsilon )}{\sigma (T_{m},p_{m})}=\frac{T}{T_{m}}\left[1-\frac{\Delta c_{p}}{\Delta s_{m}}\left(1-\frac{T}{T_{m}}\right)-\left(\frac{k_{B}\xi_{e}\ln\xi_{e}}{\Delta s_{m}}\right)\tilde{\xi }\right]+$$

$$+\frac{\varepsilon_{0}}{T_{m}\Delta s_{m}}\left(\frac{\tau_{R}}{\tau_{ns}}\right)\left[1-\exp\left(-\frac{\tau_{ns}}{\tau_{R}}\right)\right].$$

It follows as a consequence that elastic stresses not only result in a decrease of the thermodynamic force of crystallization, but also in an increase of the surface tension. Latter property is expected to enhance considerably the effect of elastic stresses on crystal nucleation and has to be accounted for in the complete resolution of the problems illustrated here with Figure 1. A detailed study of the consequences of the changes of the surface tension on crystal nucleation in highly viscous liquids is consequently a highly interesting task and will be performed in near future.

As a general conclusion we can state that the interplay of elastic stress evolution and elastic stress relaxation becomes of importance for crystal nucleation at the same conditions when deviations of the liquid from its metastable state have to be accounted for in the description of crystal nucleation. Both factors (deviations from metastable equilibrium and stresses) act consequently as a rule simultaneously. Their effects are determined by the ratio $\tau_{ns}/\tau_{R}$ of time-lag in nucleation and Maxwellian relaxation time. Elastic stress effects result in an increase of the surface tension and a decrease of the thermodynamic driving force of crystal nucleation and, consequently, in an increase of the work of critical cluster formation, i.e., they enhance the effects caused by deviations of the liquid from metastable equilibrium on cooling. Finally, we would like to note that also other mechanisms exist by which elastic stresses may affect crystal nucleation in glasses [2,106,107].

## 6. Discussion

In CNT it is assumed commonly that relaxation processes of the liquid undergoing crystallization to the respective metastable equilibrium state proceed prior to crystallization. Only in such cases, the determination of the thermodynamic driving force and the surface tension for crystallites in the metastable liquid is appropriate for the description of the thermodynamic aspects of crystal nucleation and growth. In such situation, the initial time-dependence of the nucleation rate is caused by time-lag effects connected with the establishment of a steady-state cluster size distribution up to crystallites of critical sizes as suggested first by Zeldovich and advanced then by many others [20,21,108,109].

Extending previous studies, here we developed for the first time a detailed quantitative description modeling the opposite situation, when the typical relaxation times of the liquid to the metastable liquid are large as compared with the time-lag in nucleation. As shown here, (i) such a situation occurs as a general rule when diffusion and viscosity or relaxation decouple (Figure 8). Once this is the case, (ii) the state of the liquid established initially on cooling (or by variations of other control parameters like pressure) differs from the one of the metastable liquid (Figure 3). It depends significantly on the cooling rate or, more generally, on the particular way the liquid is transferred to the state where nucleation is analyzed. (iii) These deviations will significantly affect the nucleation rate if the surface tension and the thermodynamic driving force of crystallization depend to a sufficiently large degree on the deviations from metastable equilibrium (modeled by one or a set of structural order parameters; see Figure 5, Figure 6 and Figure 7). (iv) Relaxation of the liquid proceeding slowly as compared to nucleation affects significantly nucleation and growth processes. It leads to properties as illustrated in Figure 9, Figure 10, Figure 11, Figure 12 and Figure 13. The steady-state nucleation rate varies with time but this is a quite different mechanism as compared to the change of the nucleation rate with time studied first by Zeldovich. Here it is the Maxwellian relaxation time which determines the time of approach of ultimate time-independent conditions in the relaxing liquid. (v) Elastic stress effects act in line with the effects of deviations of the liquid from the metastable state resulting in an increase of the work of critical cluster formation and a decrease of the steady-state nucleation rates (see Figure 14 and Figure 15). Their effects on nucleation increase with decreasing temperature and, as the rule, they also have to be accounted for in a comprehensive correct treatment of crystal nucleation in glass-forming liquids near and below the glass transition temperature.

**Figure 16:**
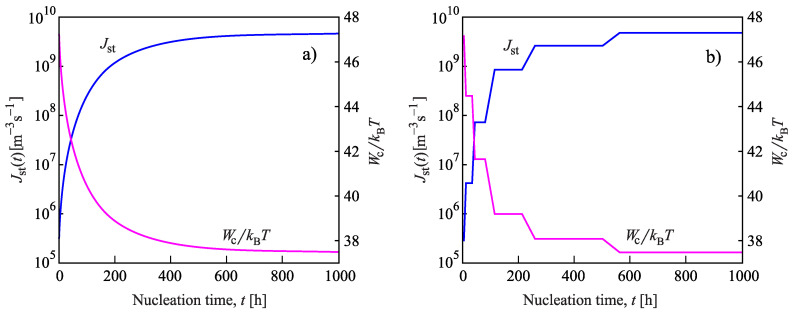
Experimental data for the time dependence of the work of critical cluster formation, $W_{c}\left(t\right)/k_{B}T$, and the steady-state nucleation rate, $J_{st}\left(t\right)$, caused by isothermal relaxation. The crystal-nucleation experiments were performed with ${\mathrm{Li}}_{2}O\cdot 2{\mathrm{SiO}}_{2}$ (L2S) at a temperature T=430 °C. The average trends are given in (**a**) in line with the phenomenological theory developed in the present study. However, there are strong indications in the detailed analysis of experimental data that the approach to metastable equilibrium is more complex. Temporarily, the liquid may be trapped in local minima of the potential energy landscape as illustrated in Figure 4d resulting in a step-wise change of the work of critical cluster formation and the steady-state nucleation rate as shown in (**b**). For the details of this and further experiments, see [38].

An experimental realization of a type of behavior as studied theoretically in the present analysis is presented in Figure 16a. It shows a time-dependence of the work of critical cluster formation and the steady-state nucleation rate being qualitatively identical to the theoretical results illustrated in Figure 12. A detailed description of the experimental data with similar curves obtained for this and other temperatures is given in [38]. However, there are strong indications in the detailed analysis of experimental data that the approach to metastable equilibrium is more complex as compared to the results shown here in Figure 12 and Figure 16a supplying us with the average general trend in the evolution of the crystallization kinetics. Temporarily, the liquid may be trapped in local minima of the potential energy landscape as illustrated in Figure 4d resulting in a step-wise change of the work of critical cluster formation and the steady-state nucleation rate. This feature observed in the experiments is shown in Figure 16b. Its analysis and further proof requires, as we believe, a detailed microscopic analysis of the effects of structural relaxation on crystal nucleation at temperatures sufficiently below the glass transition temperature.

**Figure 17:**
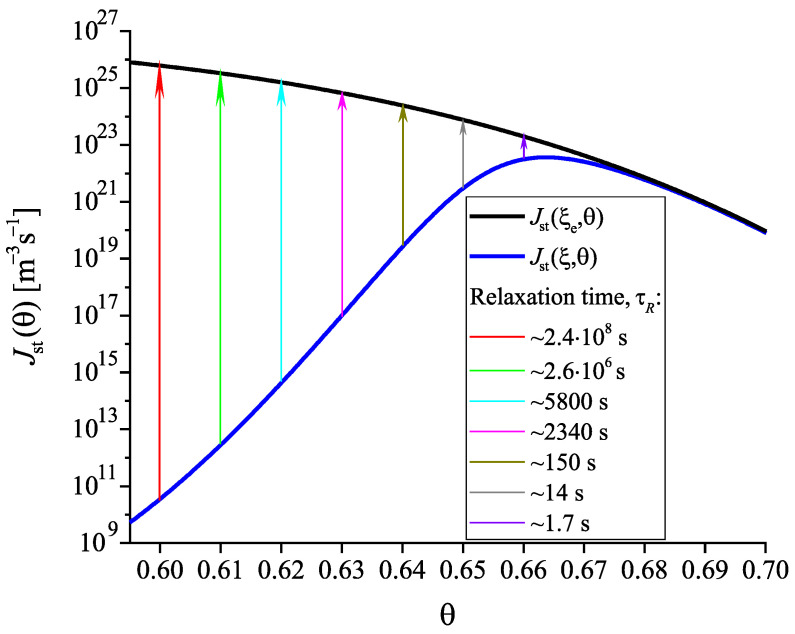
Steady-state nucleation rates, J(θ,ξ(0)), are shown as functions of temperature for the cases that the structural order parameter is equal to its value reached on cooling (for a cooling rate as employed in Figure 3 (blue curve)) and after complete relaxation to metastable equilibrium, $J(\theta ,\xi_{e})$ (black curve), representing the ultimately reached steady-state nucleation rates. In accordance with the results of analysis presented in Figure 13, the values of the Maxwellian relaxation times determine the time of evolution to metastable equilibrium. Their values for different temperatures are also given in the figure. In this illustration, elastic stress effects are not incorporated. Their account results in shifts of both curves to lower values in the temperature range where blue and black curves deviate.

In Figure 17, the steady-state nucleation rates, J(θ,ξ(0)), are given as functions of temperature for the cases that the structural order parameter is equal to its value reached on cooling (Figure 3) and after complete relaxation to metastable equilibrium, $J(\theta ,\xi_{e})$. Changes of the steady-state nucleation rates resulting from the relaxation of the structural order parameter are indicated by arrows. The characteristic times of change of the steady-state nucleation rates are determined by the value of the Maxwellian relaxation time (see also Figure 13). By this reason, the values for the steady-state nucleation rate reported in different experimental studies depend in this temperature range significantly on the duration of the experiments. For sufficiently low temperatures the metastable liquid may never be reached in typical time scales of experimental studies. However, in any case, it is always really the steady-state nucleation rate which is measured in the system for the time scales under consideration and its variations with time are not caused by time-lag effects.

A comparison of Figure 1 and Figure 17 shows that the mechanisms of crystal nucleation studied in this paper supply us with an straightforward explanation of the so-called “breakdown” of CNT at temperatures below the temperature, $T_{max}$. In cooling the liquid, near to the glass transition temperature deviations of the state of the liquid from metastable equilibrium result in nucleation rates described by the blue curve. These curves deviate from the black curve obtained in terms of CNT not accounting appropriately for such effects. Our approach and its results confirm the point of view that the mentioned anomaly can be comprehensively treated in terms of CNT and is a particular realization of even much more complex and intriguing features of crystal nucleation than commonly assumed so far (cf. [39,40]).

Two additional conclusions can be drawn from the results of our analysis illustrated in Figure 17: (i) crystal nucleation may be observed in systems at low temperatures sometimes only after sufficiently prolonged isothermal annealing (cf. [51,52,53,54,55]). Relaxation may not proceed prior to crystal nucleation in glasses because it inhibits crystal nucleation but, in contrast, it catalyzes nucleation. It leads in the course of annealing to sufficiently low values of the work of critical cluster formation resulting in sufficiently large nucleation rates which can be observed experimentally (cf. [43,44,45,46]). (ii) In polymer physics, the hypothesis has been advanced that relaxation proceeds prior to nucleation since the relaxation kinetics inhibits the formation of crystalline aggregates. Here we show that in contrast relaxation results in a decrease of the work of critical cluster formation, by this reason, it catalyzes crystal nucleation and does not inhibit it.

In the present analysis, we considered the situation that one structural order parameter is sufficient for the description of the state of the liquid. In general, a set of structural order parameters, $\left\{\xi_{j}\right\}$, may be required for that purposes [20,21,94,106,110]. This set of structural order parameters will affect thermodynamic driving force, surface tension, relaxation times, and the effective diffusion coefficient governing nucleation and growth. In such more general situation, we have to generalize Equation (11) as

(52) $$\varphi \left(T,p,\left\{x_{i}\right\};\left\{{\tilde{\xi }}_{j}\right\}\right)≅\varphi_{e}\left(T,p,\left\{x_{i}\right\}\right)+\sum_{i=1}^{f}A_{i}{\tilde{\xi }}_{i}+\sum_{i=1}^{f}\sum_{j=1}^{f}B_{ij}{\tilde{\xi }}_{i}{\tilde{\xi }}_{j}+\ldots ,\;{\tilde{\xi }}_{j}=\frac{\left(\xi_{j}-\xi_{j}^{\left(e\right]}\right)}{\xi_{j}^{\left(e\right)}}.$$

to appropriately describe, for example, the dependence of the thermodynamic driving force and the surface tension on deviations of the state of the liquid from metastable equilibrium. As a consequence, also the current steady-state nucleation rate will depend on the whole set of structural order parameters. Its evolution in time to the ultimate steady-state is determined then via

(53) $$\frac{dJ_{st}\left(T,p,\left\{x_{i}\right\};\left\{\xi_{j}\right\}\right)}{dt}=\sum_{j=1}^{f}\frac{\partial J_{st}\left(p,T,\left\{x_{i}\right\};\left\{\xi_{j}\right\}\right)}{\partial \xi_{j}}\frac{d\xi_{j}}{dt}.$$

For any of these structural order parameter statistical models have to be advanced then specifying its origin and allowing to define their equilibrium values, $\left\{\xi_{j}^{\left(e\right)}\right\}$, in dependence on temperature. Having in mind the remark of two of the founders of glass science, Davies and Jones—“*The central problem is to explain in molecular terms the way in which the glass differs from the liquid and the nature of the change from the glass to the equilibrium liquid. In view of the complexity of glass-forming substances we cannot hope for a detailed microscopic theory*” ([111], p. 374)—certain simplifying assumptions are required in order to arrive at a comprehensive but at the same time sufficiently simple and tractable theoretical description. For any of the structural order parameters we can then formulate an equation of the form of Equation (4)

(54) $$\frac{d\xi_{j}}{dt}=-\frac{1}{\tau_{j}\left(T,p,\left\{x_{i}\right\};\left\{\xi_{j}\right\}\right)}\left(\xi_{j}-\xi_{j}^{\left(e\right)}\right),\;\;j=1,2,\ldots ,f.$$

The approach of the ultimate steady-state nucleation rate will be governed by the structural reorganization of the liquid determined by the largest relaxation time, $\tau_{j}$, in the set $\left\{\tau_{j}\right\}$, j=1,2,…,f. In the phenomenological description underlying Equation (54) any of these relaxation times can be expected to model a distribution of microscopic relaxation processes determining the relaxation of the particular structural order parameter [94,110]. The analysis of the experimental data on crystal nucleation given in [38] shows that for a detailed quantitative description, at least, two structural order parameters are required.

Finally, we would like to underline once again that in the temperature range where deviations of the liquid from its metastable initial state have to be accounted for in the description of nucleation ($(\tau_{ns}/\tau_{R})\ll 1$) also the interplay of evolution of elastic stresses and stress relaxation affects considerably crystal nucleation. Accounting for such stress effects, the black curve in Figure 17 will never be reached even for infinite relaxation times. As shown in Section 5, a correct description of the effect of elastic stresses in crystallization in the transition of a liquid from low-viscosity fluid to high-viscosity fluid and/or solid requires the fulfilment of this inequality ($(\tau_{ns}/\tau_{R})\ll 1$). Once one has to expect that sufficiently below $T_{g}$ the glass behaves as a solid, always this inequality will be realized with decreasing temperature. This consequence leads also to the conclusion that deviations of the liquid from its metastable state have to be accounted for, as a rule, if crystal nucleation is described near and below $T_{g}$. A detailed analysis of the effect of elastic stresses on crystallization has been performed recently by us in [19] as one possible method of resolution of the low temperature anomaly in the theoretical description of crystal nucleation. The account of the effect of elastic stresses on the surface tension, as described in the present paper, will increase its influence on crystal nucleation. A detailed analysis of the consequences of stress effects on the surface tension and its consequences will be performed in a future analysis.

Note also that, in general, self-consistency corrections have to be accounted for in the determination of the work of critical cluster formation, as discussed in detail in one of our preceding papers [13]. Such corrections will, however, not change the general results derived here and by this reason are omitted in the present paper for clarity of the presentation. As shown in the cited paper, self-consistency corrections do not affect the results of theoretical analysis if the process is studied by solving the set of kinetic equations modeling crystal nucleation and growth in the framework of CNT. This model can be used also in the analysis of the spectrum of problems analyzed in the present paper. In addition to the conventional procedure, one has to add then Equations (4) and (5) to model the change of the structural order parameter with time and/or temperature and to take into account changes of the kinetic coefficients (Equation (39)) accounting for stress evolution and stress relaxation. Latter procedure can be performed employing the results outlined in [104,105]. Utilizing this set of kinetic equations one can arrive at a more detailed description of nucleation and growth accounting appropriately for effects of glass transition, structural relaxation, and the interplay of stress development and stress relaxation at any desired path of change of temperature (or other external control parameters). In particular, one can describe in such an approach the influence of preformed nuclei on crystal nucleation kinetics in glasses near and below the glass transition temperature as studied, for the case of soda-lime-silica glasses, experimentally in [112,113]. The general conclusions derived in the present analysis will remain, of course, unchanged in such more detailed description.

## 7. Conclusions

1. On cooling, a liquid is transferred, in the glass transition range and below it, into a thermodynamically non-equilibrium state, the glass. Therefore, its properties become different from the properties at the corresponding (relaxed) metastable equilibrium state. As a consequence, the main thermodynamic and kinetic parameters determining the rate of crystal nucleation become dependent on such deviations from metastable equilibrium. These deviations result, in particular, in changes of the theoretical estimates of the steady-state nucleation rates and the time-lag in nucleation. The glass transition temperature and the properties of the glass depend on cooling and heating rates or, more generally, on the path the system is transferred into the state where nucleation is studied. Consequently, accounting for deviations of the state of the liquid from metastable equilibrium, its dependence on cooling and heating rates or the way the system is transferred into the respective state has to be determined.
2. Experiments on the determination of the steady-state nucleation rate and the time lag in nucleation are normally performed by transferring the liquid into the desired initial state at moderate cooling rates. At such process conditions, the glass transition takes place at a temperature $T_{g}^{\left(T\right)}$, defined by Tammann as the temperature at which the liquids Newtonian viscosity, η, is equal to $\eta \left(T_{g}^{\left(T\right)}\right)≅10^{12}$ Pa s. At such typical experimental conditions, the glass transition temperature and the temperature of the maximum crystal nucleation rate, $T_{max}$ and $T_{g}^{\left(T\right)}$, are close, $T_{max}≊T_{g}^{\left(T\right)}$. Consequently, the hypothesis can be advanced that specific features of crystal nucleation occurring at the glass transition temperature and below it may be the origin for the problems in the theoretical interpretation of experimental results on steady-state nucleation rates near and below the maximum of the steady-state nucleation rate.
3. As shown here, deviations from metastable equilibrium (caused by cooling and the resulting transfer of a relaxed supercooled liquid into a glassy state) always result in higher values both of the thermodynamic driving force of crystallization and the surface tension as compared to phase formation in a metastable liquid. Consequently, isothermal annealing processes in the course of nucleation (relaxation of the glassy state towards metastable equilibrium) are accompanied by a decrease of both the thermodynamic driving force of crystallization and the surface tension. As one of the consequences of these processes, isothermal relaxation leads, as a rule, to a decrease of the work of critical cluster formation and an increase of the steady-state nucleation rates.
4. The effect of described above deviations of the liquid from metastable equilibrium on crystal nucleation depends on the ratio of the characteristic time scales of nucleation, 〈τ〉, and relaxation, $\tau_{R}$. In the range of temperatures of relevance for the present study, $T⪅1.2T_{g}^{\left(T\right)}$, the average time of formation of the first supercritical nucleus, 〈τ〉, is approximately equal to the time-lag in nucleation, $\langle \tau \rangle ≅\tau_{ns}$. If the capillarity approximation and the Stokes–Einstein–Eyring equation are assumed to be fulfilled, then the inequality $\tau_{R}\ll \tau_{ns}$ is always satisfied and relaxation is always completed prior to crystal nucleation. However, these two assumptions are known to be of limited validity. If they are removed, both cases $\tau_{R}\ll \tau_{ns}$ and $\tau_{R}\gg \tau_{ns}$ may be realized and crystallization may proceed prior to relaxation. Provided the latter condition, $\tau_{R}\gg \tau_{ns}$, is fulfilled, then nucleation proceeds concomitantly with relaxation. For its description, expressions for the thermodynamic driving force and the surface tension have to be employed where deviations of the state of the liquid from metastable equilibrium are accounted for. Consequently, at such conditions, they may be responsible for the deviations of theoretical predictions of steady-state nucleation rates from experimental data as illustrated in Figure 1. The validity of this hypothesis is proven in the present study.
5. Provided the condition $\tau_{R}\gg \tau_{ns}$ is fulfilled, moderate variations of the state of the liquid with time in the course of nucleation and growth take place. As one consequence, steady-state nucleation rates may change also slowly with time. The characteristic time-scales of such variations of the state of the liquid and the steady-state nucleation rate are determined by the Maxwellian relaxation times. They may be very large, so that they are frequently not reached in experimental analyses of nucleation below $T_{g}$. Anyway, the experimental studies performed and exhibiting the “breakdown of CNT” problem represent measurements of the steady-state nucleation rates for the time interval where they are performed. In this sense, they remain correct and they are described by CNT, if, as shown here, deviations of the state of the liquid from metastable equilibrium are accounted for. In those measurements, the liquid had not reached metastable equilibrium and, as a consequence, the ultimate steady-state nucleation regime of nucleation was not established. In fact, at sufficiently deep supercooling, the metastable liquid may not be reached at all at reasonable laboratory time scales. Consequently, the computations employing the method outlined in the present paper provide an independent confirmation that the low temperature anomaly in silicate glasses is a particular realization of even more complex, intriguing features of crystal nucleation than commonly assumed so far.
6. Since relaxation is connected with an increase of the steady-state nucleation rates, crystal nucleation may be observed in systems at low temperatures sometimes only after sufficiently prolonged isothermal annealing. Relaxation does not occur prior to nucleation in such cases because it inhibits crystal nucleation but, in contrast, it leads to sufficiently low values of the work of critical cluster formation resulting in sufficiently large nucleation rates which can be observed experimentally.
7. The condition, $\tau_{R}\gg \tau_{ns}$, which has to be fulfilled that glass transition and relaxation may affect nucleation leads simultaneously to the requirement that elastic stresses may become of significant importance for the specification of the thermodynamic driving force and the surface tension. Elastic stresses result in a decrease of the thermodynamic driving force and, as shown here for the first time, in an increase of the surface tension. In this way, they act in the same direction as deviations of the state of the liquid from equilibrium leading to an increase of the work of critical cluster formation. Experimental data on crystal nucleation near and below the glass transition are consequently always affected by both factors—deviations from metastable equilibrium and elastic stresses—once the inequality $\tau_{R}\gg \tau_{ns}$ holds.
8. Finally, the present analysis gives a new theoretical interpretation of the possibility of existence of crystal nucleation flashes during heating. They are expected to be of significant importance, in particular, for the correct description of cold crystallization, a phenomenon widely discussed in polymer physics. This highly interesting consequence of the formalism presented here will be analyzed in detail in a future study. However, this is only one in the wide spectrum of its possible applications to the description of crystal nucleation and growth processes where the advanced here method of theoretical analysis is believed to lead to new insights.

## Appendix A. Some Features of the Lattice-Hole Model Employed in the Present Analysis

The thermodynamic functions of the liquid are described in the framework of the lattice-hole model used in the present analysis by the sum of contributions resulting from the thermal motion of the molecules of the liquid supplemented by configurational contributions described by the structural order parameter, ξ. The configurational contribution to the molar volume (or the excess volume) is given by [20,21]

(A1) $$V_{conf}≅N_{A}v_{0}\left(p,T\right)\xi .$$

Here $N_{A}$ is the Avogadro number. The configurational contribution to the enthalpy, $H_{conf}$, of one mole of the liquid is described in the framework of this lattice-hole model via the molar heat of evaporation, $\Delta H_{ev}\left(T_{m}\right)$, of the liquid at the melting temperature as

(A2) $$H_{conf}=\chi_{1}\Delta H_{ev}\left(T_{m}\right)\xi .\;\Delta H_{ev}\left(T_{m}\right)≅\chi_{2}RT_{m}\;\mathrm{with}\;\chi_{2}=20.$$

The parameter $\chi_{1}$ will be determined later.

The configurational part of the entropy per mole is described in this model via the conventional mixing term

(A3) $$S_{conf}=-R\left(\ln\left(1-\xi \right)+\frac{\xi }{1-\xi }\ln\xi \right).$$

Employing a truncated Taylor expansion of $S_{conf}\left(\xi \right)$ in the vicinity of $\xi =\xi_{e}$, the difference $S_{conf}\left(\xi \right)-S_{conf}\left(\xi_{e}\right)$ can be written approximately as

(A4) $$S_{conf}\left(\xi \right)-S_{conf}\left(\xi_{e}\right)≅-R\ln\xi_{e}\left(\xi -\xi_{e}\right)=-R\left(\xi_{e}\ln\xi_{e}\right)\tilde{\xi },$$

where $\tilde{\xi }$ is defined by Equation (10).

With H=U+pV, we obtain for the configurational contribution to the internal energy the relation

(A5) $$U_{conf}=\left[\chi_{1}\Delta H_{ev}\left(T_{m}\right)-pv_{0}\left(p,T\right)\right]\xi .$$

Finally, employing the definition of Gibbs’ free energy, G=U−TS+pV, we arrive at

(A6) $$G_{conf}=\chi_{1}\Delta H_{ev}\left(T_{m}\right)\xi +RT\left(\ln\left(1-\xi \right)+\frac{\xi }{1-\xi }\ln\xi \right).$$

The equilibrium value of the structural order parameter, $\xi =\xi_{e}$, is determined via the relation ${\left(\partial G_{conf}/\partial \xi \right)}_{p,T}=0$. With Equations (A2) and (A6), we obtain the following result

(A7) $$\frac{{\left(1-\xi_{e}\right)}^{2}}{\ln\xi_{e}}=-\frac{1}{\chi }\left(\frac{T}{T_{m}}\right)\;\mathrm{where}\;\chi =\chi_{1}\chi_{2}.$$

Knowing the value of $\chi_{2}$ (cf. Equation (A2)), we determine the value of the parameter $\chi_{1}$ demanding that at $T=T_{m}$ the value of $\xi_{e}$ should be approximately equal to 0.05 (corresponding to experimentally observed density differences between liquid and crystal at the melting temperature, $T_{m}$ [20,21]). In the computations performed here we set $\chi_{2}=20$ and $\chi_{1}=0.166$ resulting in χ=3.32.

As should be the case, in the vicinity of the state of configurational equilibrium (defined via the condition ${\left.\left(\partial G_{conf}/\partial \xi \right)\right|}_{p,T,\xi =\xi_{e}}=0$), we obtain from Equation (A6) after performing a truncated Taylor expansion the result.

(A8) $$G_{conf}\left(p,T,\xi \right)≅G_{conf}\left(p,T,\xi_{e}\right)+\frac{1}{2}{\left.\left(\frac{\partial^{2}G_{conf}}{\partial \xi^{2}}\right)\right|}_{p,T,\xi =\xi_{e}}{\left(\xi -\xi_{e}\right)}^{2}.$$

The value of $G_{e}^{\left(2\right)}=\left(\partial^{2}G_{conf}/\partial \xi^{2}\right){|}_{\xi =\xi_{e}}>0$ at equilibrium can now be easily calculated based on Equations (A6) and (A7). For the considered case of small values of ξ, we get as a sufficiently correct estimate [67].

(A9) $$G_{e}^{\left(2\right)}≅\frac{RT}{\xi_{e}}.$$

Equations (A4) and (A9) are used in the present analysis for the generalization of the expressions for the thermodynamic driving force and the surface tension in the description of crystal nucleation.
